# Characterization and transcript profiling of the pectin methylesterase (PME) and pectin methylesterase inhibitor (PMEI) gene families in flax (*Linum usitatissimum*)

**DOI:** 10.1186/1471-2164-14-742

**Published:** 2013-10-30

**Authors:** David Pinzón-Latorre, Michael K Deyholos

**Affiliations:** 1 Department of Biological Sciences, University of Alberta, Edmonton, AB T6G 2E9, Canada

**Keywords:** Flax, Pectin methylesterase, Fiber, Expression analysis, Phylogenetics, PME, PMEI

## Abstract

**Background:**

Pectin methylesterases (PMEs) catalyze the demethylesterification of homogalacturonans in the cell wall; their activity is regulated in part by pectin methylesterase inhibitors (PMEIs). PME activity may result in either rigidification or loosening of the cell wall, depending on the mode of demethylesterification. The activity of PMEs in the middle lamella is expected to affect intrusive elongation of phloem fibers, and their adhesion to adjacent cells. Length and extractability of phloem fibers are qualities important for their industrial uses in textiles and composites. As only three flax PMEs had been previously described, we were motivated to characterize the PME and PMEI gene families of flax.

**Results:**

We identified 105 putative flax PMEs (LuPMEs) and 95 putative PMEIs (LuPMEIs) within the whole-genome assembly. We found experimental evidence for the transcription of 77/105 LuPMEs and 83/95 LuPMEIs, and surveyed the transcript abundance of these in 12 different tissues and stages of development. Six major monophyletic groups of LuPMEs could be defined based on the inferred relationships of flax genes and their presumed orthologs from other species. We searched the LuPMEs and LuPMEIs for conserved residues previously reported to be important for their tertiary structure and function. In the LuPMEs, the most highly conserved residues were catalytic residues while in the LuPMEIs, cysteines forming disulfude bridges between helices α2 and α3 were most highly conserved. In general, the conservation of critical residues was higher in the genes with evidence of transcript expression than in those for which no expression was detected.

**Conclusions:**

The LuPMEs and LuPMEIs comprise large families with complex patterns of transcript expression and a wide range of physical characteristics. We observed that multiple PMEs and PMEIs are expressed in partially overlapping domains, indicative of several genes acting redundantly during most processes. The potential for functional redundancy was highlighted also by the phylogenetic analyses. We were able to identify a subset of PME and PMEIs that appeared particularly relevant to fiber development, which may provide a basis for the improvement of key traits in industrial feedstocks and a better understanding of the physiological roles of PMEs and PMEIs in general.

## Background

Pectins are complex polysaccharides present in the plant cell wall and in the middle lamella and are dynamically modified by pectin methylesterases (PMEs). The PME gene family was first described by Richard et al. [1], and later classified in the Carbohydrate Active Enzymes database (CAZy) as class 8 of the carbohydrate esterases (EC 3.1.1.11) [2]. In current models, pectins are synthesized in the Golgi complex as highly methylesterified polymers (e.g. homogalacturonan, HG) that are secreted to the cell wall. Once in the cell wall, PMEs catalyze the demethylesterification of HG, which generates negatively charged carboxyl groups. If demethylesterification occurs on contiguous sugar residues (i.e. blockwise demethylesterification), Ca^2+^ bonds can form between pectin molecules, thereby rigidifying the cell wall. Conversely, if the demethylesterification occurs on non-contiguous sugars (i.e. random demethylesterification), the molecule becomes a substrate for pectin degrading enzymes, leading to cell wall loosening [3,4]. The activity of the PMEs is regulated by pectin methylesterase inhibitors (PMEIs) [5], which bind to the active site of the PME, generating a 1:1 complex [6,7]. PMEs are classified as either Type-1 PMEs (i.e. those with a pro-region, similar to the PMEI domain), or Type-2 PMEs (no pro-region). In Type-1 PMEs, the pro-region and the PME domain are translated as part of the same protein and then, in the Golgi complex, as a pre-requisite for secretion to the cell wall, the pro region is removed by a subtilisin-like protease [8].

The bast (phloem) fibers of flax (*Linum usitatissimum)* are valued industrially for their length and strength. Extraction of high quality fibers requires retting, a process by which stems are exposed to the action of microbes that degrade the middle lamella and so, facilitate separation of fibers from surrounding tissues. Flax fibers grow from the shoot apex intrusively after a very short period of coordinated growth [9]. During intrusive elongation, fibers first penetrate the middle lamella between adjacent cells, and subsequently generate new contact interfaces. Both of these processes presumably influence fiber length and the efficiency of retting, and are dependent on the activity of PMEs.

Different varieties of flax are grown for either fibers or for seeds (i.e. linseed) [10]. Although stems of linseed varieties contain fiber, these fibers are not harvested, because of relatively low fiber yield and the difficulty of retting in the environments where linseed is typically grown. A better understanding of PMEs is therefore important to the development of dual-purpose flax, in which both fibers and seeds can be utilized from a single variety.

Three PMEs have been previously characterized in flax: LuPME1, LuPME3, and LuPME5 [11-14]. These are all Type-1 PMEs. Al-Qsous *et al.*[13] reported that in 2 dpg (days past germination) hypocotyls, transcript abundance of LuPME5 is higher in the apical region, while LuPME3 transcript abundance is higher in the basal region. Also, LuPME5 has the highest transcript abundance of the three characterized LuPMEs in hypocotyls. The highest transcript abundance of LuPME3 in seedlings is in the roots [11]. Here, we expand on these studies and present an analysis of the complete family of PMEs and PMEIs in flax, based on the recently assembled whole genome sequence of the linseed variety CDC Bethune [15]. A specific objective of this research is to identify PMEs that are expressed during stages of fiber development that are likely to influence the industrially relevant properties of flax bast fibers.

## Methods

### Annotation of PME and PMEI domain in flax and other species

Predicted proteins that contained PME (PF01095) and/or PMEI (PF04043) domains were identified from the whole genome shotgun (WGS) assembly of flax [15] (version 1.0) using default parameters in hmmsearch/PfamScan [16]. The predicted proteins from the flax WGS assembly were also aligned to previously described PMEs and PMEIs from Arabidopsis obtained from TAIR [17], using BLASTp. All of the LuPMEs/LuPMEIs that were identified by BLASTp to Arabidopsis were also identified by HMM-alignment to the PFAM domains. Predicted flax proteins that had both a PMEI and PME domain were designated Type-1 PMEs, and proteins with a PME domain (but no PMEI domain) were designated Type-2 PMEs. Genes with questionable PFAM annotations (i.e. significant PME and/or PMEI domain but low e-value; low coverage of the domain; more than one PME or PMEI domain; an extra domain different from PME or PMEI), and genes that were adjacent on scaffolds of the WGS assembly were manually curated, which included reanalysis of their predicted gene structures by submitting their genomic sequence (i.e. the predicted gene plus 1000 bp up and downstream) to the Augustus web server [18]. The Augustus *A. thaliana* gene model parameters were used for the gene reannotation, in combination with any ESTs that aligned to the prediction region (95% identity and 90% coverage) [19] as well as unpublished RNAseq reads (http://www.onekp.com/, version April 25 2013).

### PMEs and PMEIs in other plants

An hmmsearch using PFAM domains PF01095 (Pectinesterase) and PF04043 (PMEI) was conducted with default parameters on transcripts deposited in Phytozome (version 9.1). To determine the statistical significance of the presence of the PME domain, all the protein sequences that had the domain were retrieved, and these were searched again against PFAM using batch search. For the putative PMEIs, protein sequences that had a PMEI domain but not a PME domain were obtained and then searched again on PFAM to establish the statistical significance of the predicted domains and confirm the absence of a PME domain.

### Primer design for qRT-PCR

The Universal ProbeLibrary Assay Design Center (Roche) was used to design specific primers and probes for each gene. Groups of 10 closely related genes were submitted in batches for the design of specific primers and Roche UPL probes. The specificity of primers was evaluated by BLASTn alignment of the primers against the complete predicted transcriptome and the entire genome assembly. All primer pairs were designed so at least one primer of each pair had three or more mismatches to any off-target gene, near the 3’ of the primer. For those genes for which a specific primer could not be designed, a primer common to two PME or PMEI genes was used. The list of primers can be accessed in Additional file 1: Table S1.

### Tissues for quantitative real time PCR using a 96.96 Dynamic array

RNA was obtained from 12 different tissues from three biological replicates. Each biological replicate was assayed independently. Five of the tissues/organs (shoot apex (SA), leaves (L), roots (R), early cortical peel (ECP), and early fibers (EF)) were collected from vegetative stage plants 1 month after germination; the other seven tissues (senescent leaves (SL), xylem (X), late cortical peel (LCP), late fibers (LF), flower buds (FB), flowers (F), and green bolls (B)) were collected from plants 2 months after germination, at the green capsule stage. The cortical peel, xylem, and fiber tissues were obtained from the first 15 cm of the plant from the hypocotyls to the top. The shoot apex tissue corresponds to the top 2 cm of the plant. A phenol/chloroform based method was used for extraction of RNA, with subsequent treatment with DNAse. 5 ng of RNA were used to synthesize the cDNA for the 96.96 dynamic array (Fluidigm Corporation, CA, USA). The cDNA was tested for genomic DNA contamination by PCR using a set of primers flanking an intron.

A total of 12.5 ng of cDNA were used for the pre-amplification reaction containing 50 nm of each primer pair (a pool consisting of 89 primers for the PME and/or PMEI genes plus 3 endogenous controls GAPDH, ETIF1, ETIF5A [20], and 1x TaqMan PreAmp Master Mix (Applied Biosystems) in a final volume of 10 μL. The following thermal cycles were followed: 1 cycle: 95°C 10 min; 14 cycles: 95°C 15 seconds, 60°C 4 min. The pre-amplified product was diluted 1:5 and the pre-amplification reaction was tested doing a pass/fail test with GAPDH endogenous control primers, verifying that the C_T_ value was close to 20.

Primer and Roche UPL probe mix (“promer”) was prepared by mixing 2 μl of a 20 μM mix of both primers for each 1 μL of the respective probe (10 μM stock). The “promer” was tested with an equimolar mixture of cDNA from all the tissues (except fiber, which are included in the cortical peels), if it did not work new primers were designed, if the new primers did not work, it was presumed to be not expressed and the primers were used regardless, and run in the 96.96 dynamic array (Fluidigm Corporation, CA, USA).

Fluidigm 96.96 control line fluid was used to prime the fluidics arrays with the 136x chip prime script. Then the appropriate inlets were loaded with the different assays and sample mixes. The three biological replicates for each tissue were each placed in three different positions on the array for three technical replicates each.

The manufacturer’s protocol was followed to prepare the assay and sample mixes. The assay inlets contained a 6.5 μL assay mixture containing 1x DA assay loading reagent and 2 μL of the respective “promer” (primer + probe) mixture for each inlet. The sample inlets contained 1x TaqMan® Universal PCR Master Mix, No AmpErase® UNG (Applied Biosystems PN 4324018), 1x DA sample loading reagent (Fluidigm PN 85000735), and 2.5 μL of the respective preamplified sample.

Once the samples and assays inlets were loaded, the 136x load mix script was executed to load the samples and assays. Once loaded, the chip was moved to the Biomark instrument and the following thermal cycles were executed: 1 cycle: 95°C 10 min; 40 cycles: 95°C 15 seconds, 60°C 1 min.

### Analysis of 96.96 dynamic array results

Only those wells with a quality score of ≥ 0.65 were used in further analyses. The mean of the technical replicates was calculated. Then the delta-C_T_ was obtained by calculating the geometric mean of the endogenous controls for the given tissue/biological-replicate, and subtracting that value to the C_T_ of the gene at that tissue/biological-replicate. Subsequently, the mean and the standard error of the delta-C_T_ of the three biological replicates were calculated.

### EST and RNAseq data mapping

The EST and assembled RNAseq data (Deyholos *et al.*, manuscript in preparation) were mapped against the PME and PMEI CDS sequences, using the read mapping tool, on the CLC Genomics Workbench 6.0.1, with the default parameters, except for the length fraction (0.8), and the similarity fraction (0.9 or 0.98).

### Signal peptide, transmembrane domain, and protein subcellular localization predictions

SignalP 4.0 was used to search for signal peptides [21] Transmembrane domains were predicted using TMHMM v.2.0 [22]. The protein subcellular localization was predicted using WoLF PSORT and Plant-mPLoc [23,24].

### Cleavage site prediction

Proteolytic cleavage sites were predicted using a protease recognition pattern described by Pelloux *et al.* and Wolf *et al. *[4,8]. The motif [RKQ][RKEHLN][LDMI][LMAKR] was searched in the Type-1 PME proteins using “Protein Pattern Find” at http://www.bioinformatics.org/. These sites were also identified visually on a ClustalW multiple alignment of the protein sequences of sequences in the same phylogenetic group. This allowed us to confirm the motifs found with the web tool, and also to identify possible novel cleavage recognition motifs, by comparison of the aligned sequences with known motifs.

### Isoelectric point

The predicted isoelectric point of the complete and the mature proteins (i.e. after signal peptide and/or cleavage site removal) was calculated using Vector NTI 10 [25].

### Phylogenetic analysis

Phylogenetic relationships among the PMEs and PMEIs from flax, *Manihot esculenta, Ricinus communis, Populus trichocarpa,* and *Arabidopsis thaliana* were inferred as follows. The PME and PMEI protein sequences from *M. esculenta, R. communis, P. trichocarpa,* and *A. thaliana* were extracted from Phytozome (version 9.1) using the PFAM identifiers PF04043 (for PMEI) and PF01095 (for PME) in a keyword-based ontology search. Alignments for the complete PMEs and PMEIs proteins of these four species plus flax, and for the LuPMEs and LuPMEIs proteins of flax alone, were constructed using MUSCLE [26]. The alignments were used to first determine the substitution model that best described the evolutionary process of each set of proteins, using ProtTest [27], and then these models were used to construct maximum likelihood trees using GARLI [28] under the CIPRES web interface [29], with 100 bootstraps and 2 search-replicates. The result of the analysis in ProtTest showed that the model of evolution that best fit the set of genes for LuPMEs was WAG + I + G + F, and the same model was obtained for the LuPMEIs. For the analysis of the PMEs and PMEIs in all the analysed species, the best model was WAG + G + F. To estimate the divergence time of presumptive paralogs (Ks) we aligned the nucleotide sequences of the LuPMEs and the LuPMEIs, and then we used MEGA5 [30] to determine genetic distance, for which we used the Kimura 2-parameter model [31] with the pairwise deletion option, and then calculated the divergence time using t = K/2r, where t is time, K is the genetic distance, and r is the substitution rate, either 1.5 × 10–8 [32] or 8.1 × 10–9 [33].

### Conserved residues

The presence of the most important residues for the protein activity was established based on the structural analysis done for the PME [7] and PMEI [6,7]. For PME 11 important residues were searched: six active sites with conserved aromatic residues, three catalytic residues, and two protein stabilizers. For PMEI, 33 important residues were analysed, including residues interacting with the active sites of the PME, residues responsible for disulfide bridging, and several residues responsible for maintaining the structure of the protein.

## Results and discussion

### Annotation of LuPMEs and LuPMEIs

**Figure 1:**
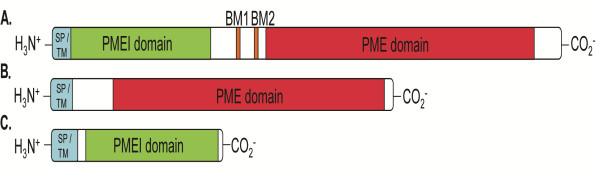
**Representatives of the three types of proteins classified in this report. A**. Examples of Type-1 PME: LuPME1; **B**.Type-2 PME: LuPME10; **C**. PMEI: LuPMEI1. SP: Signal peptide. TM: Transmembrane domain. BM: Binding motif.

We identified 105 putative LuPMEs and 95 putative LuPMEIs (Additional file 2) by searching predicted transcripts of the flax whole-genome assembly (version 1.0) [15] for the PFAM domains Pectinesterase (PF01095) and PMEI (PF04043) [16]. Independent alignment of the *Arabidopsis thaliana* PME and PMEI families [34] to the flax genome did not identify any additional flax genes other than those identified by the PFAM domain alignment. Among the predicted LuPMEs, 60 were Type-1 (i.e. encoding both a PMEI (PF04043) and PME (PF01095) domain [3]), and 45 were Type-2 (i.e. encoding a PME domain, but no PMEI domain [3]; Figure 1). Only one of the genes (LuPME89) contained an additional PFAM domain other than a PME or PMEI domain. This was a zf-RING_2 domain (PF13639).

### Detection of PMEs and PMEIs in other plants

To identify putative PMEs and PMEIs in species other than flax, primary transcripts in the Phytozome v9.1 database were searched for the presence of a PME or PMEI domain. The number of predicted PMEs and PMEIs in each species was compared as a proportion of all proteins predicted for each species (Additional file 3: Figure S1). The proportion of PMEs (0.25%) and PMEIs (0.22%) in flax was similar to the average proportion in the Malpighiales species sampled, i.e. 0.23% and 0.16%, respectively. Among the plants analyzed, *Mimulus guttatus,* followed by *Capsella rubella* had the highest proportion of PMEs 0.30% and 0.29%, respectively. Meanwhile *C. rubella* and *Arabidopsis thaliana* had the highest proportion of PMEIs 0.31% and 0.27%, respectively. The proportion of PMEs was diminished significantly in the grasses as compared to other angiosperms (t-test p < 0.01). On average the angiosperms (not including grasses) had 0.22% PMEs as a proportion of the total predicted gene number, while grasses had 0.12%.

### Transcript expression profiling

We examined transcript expression data to determine whether each predicted LuPME and LuPMEI was expressed, and if so, under what circumstances. Data sources for this analysis included qRT-PCR experiments (described below), published microarray data (Fenart *et al.*[35]), published flax ESTs (Venglat *et al.*[36]; NCBI), and unpublished Illumina RNAseq read data from the flax shoot apical meristem (Deyholos et al., manuscript in preparation), and from the developing flax stem (One Thousand Plants Consortium, manuscript in preparation).

### qRT-PCR using a 96.96 dynamic array

**Figure 2:**
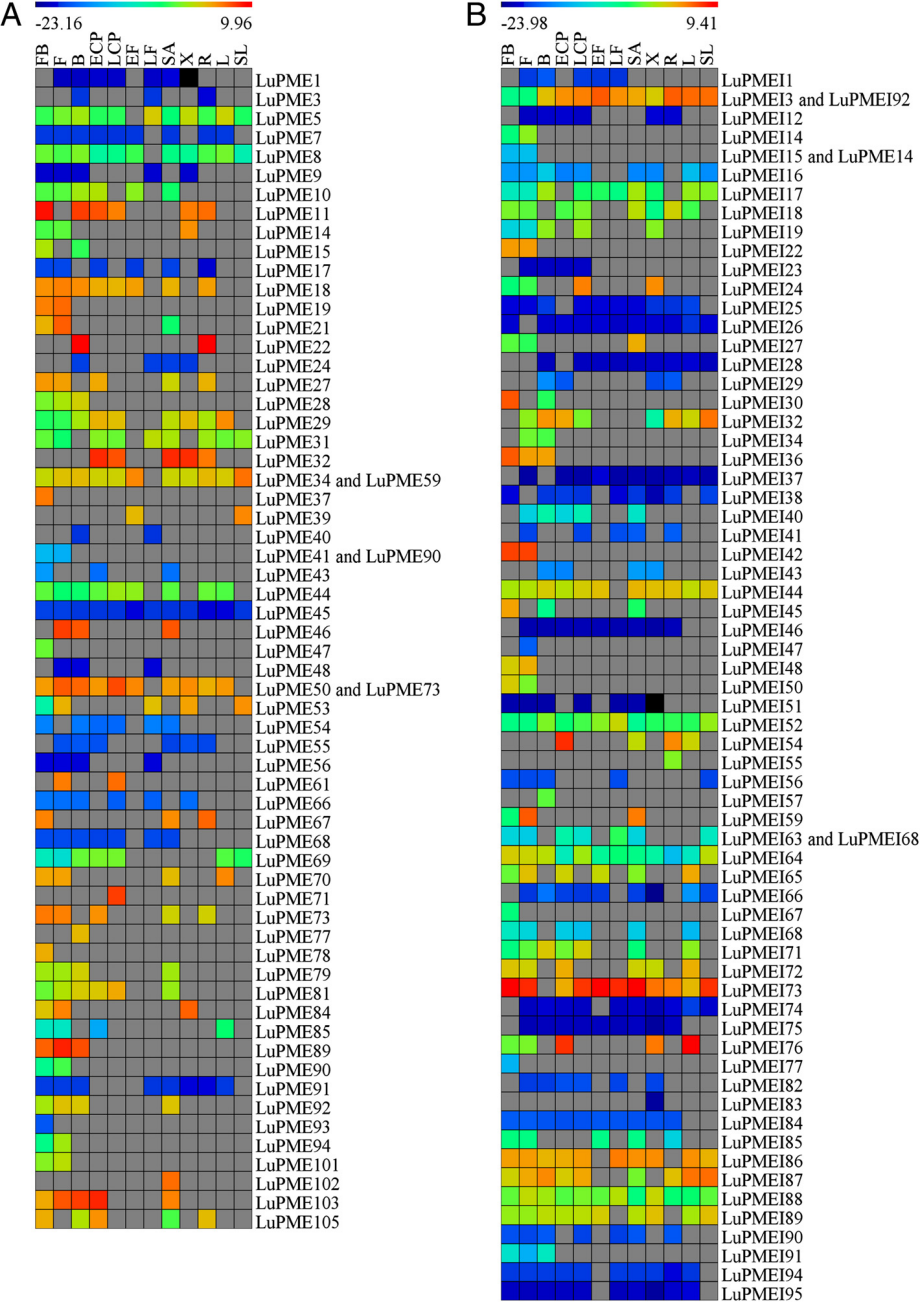
**Heat map of transcript abundance of PMEs and PMEIs in different tissues.** PMEs are shown in **(A)** and PMEIs are shown in **(B)**. Delta-C_T_ (C_T_ of gene minus C_T_ of geometric mean of the endogenous controls). The color of the cell represents transcript abundance. Gray cells indicate no transcripts were detected. When two different genes appear in the same row it means one set of primers was used as a common assay for both genes. FB: Flower buds; F: Flowers; B: Green boll; ECP: Early cortical peels; EF: Early fibers; LF: Late fibers; SA: Shoot apical meristem; X: Xylem; R: Roots; L: Leaves; SL: Senescent leaves.

We used a Fluidigm 96.96 microfluidic array to conduct qRT-PCR on 12 different tissues of flax. Five of the tissues (shoot apex (SA), leaves (L), roots (R), early cortical peel (ECP), and early fibers (EF)) were collected from vegetative stage plants 1 month after germination; the other seven tissues (senescent leaves (SL), xylem (X), late cortical peel (LCP), late fibers (LF), flower buds (FB), flowers (F), and green bolls (B)) were collected from plants 2 months after germination, at the green capsule stage. We were able to design gene-specific primers for 102 out of the 105 predicted PMEs. Transcripts of 62/102 PME genes (60.8%) were detected in at least one of the tissues (Figure 2A), with a minimum Fluidigm 0.65 quality score and in at least 2 out of 3 biological replicates. 40/102 predicted PMEs (39.2%) could not be detected in any of the tissues assayed by qRT-PCR. However, transcripts of 6/40 of these genes could be found among public ESTs collections (80% coverage and 98% identity), and an additional 8/40 genes could be identified among reads from RNAseq transcript profiling experiments (manuscript in preparation) (Tables 1 and 2). Finally, one of the three predicted PMEs for which qRT-PCR was not attempted was also detected by RNAseq. The unigenes assembled by Fenart *et al.*[35] from ESTs of a flax fiber variety were also queried. Out of the 20 unigenes (16 PMEs and 4 PMEIs) that mapped with 80% coverage and 98% identity to the predicted LuPMEs listed in Tables 1 and 2, 14 PMEs and 3 PMEIs were detected within the Fluidigm qRT-PCR array, while all were identified by either the EST collections or the RNAseq data. In total we were therefore able to find experimental evidence for the transcription of 77/105 predicted PMEs (Tables 1 and 2).

**Table 1:** Major features of type-1 LuPMEs

**LuPME**	**Mature protein**			**Binding motif for cleavage**			**Subcellular localization**				**Conserved residues**			**Tree**	**Expression**						
	**AA**	**Kda**	**pI**	**BM1**	**BM2**	**positions**	**sp**	**tm**	**WolF PSORT**	**Plant-mPLoc**	**as**	**st**	**cr**	**Group**	**oe**	**fl**	**uf**	**ev**	**en**	**rs**	**rsa**
1	333	34.9	8.82	RKLL	RKLL	203-223	+	-	ch, m	cw	6	2	3	D	+	+				S3	+
3	320	34.4	9.77		RRLL	235	+	+	ch, v, ex	cw	6	2	3	D	+	+					+
5	319	34.6	9.53		RRLL	234	-	+	pl, g_pl, er, g	cw	6	2	3	D	+	+	1	S, SP, F	5	All	+
6	322	35.5	7.18	RKLL	RRVL	202-217	+	-	ch, n, pl	cw	5	2	3	C	+	-					+
7	322	35	6.6	RKVA	RRLL	235-259	+	-	ch, v, n	cw	6	2	3	D	+	+	1	S	2	All	+
8	364	40.1	8.66	RKLL	RRLL	263-286	-	+	n, cy, v, er, m, pl	cw	6	2	3	B	+	+	1	S	2	S3 and 4	+
9	322	35.6	9.6	RRLL	RKLL	224-242	+	+	ex, er, v, g, ch, n, cy	cw	5	2	3	C	+	+		F	34		+
11	593	64.1	8.46				-	+	ch, m	cw	6	2	3	A	+	+	1	ES, HE, TE,GS	4	All	+
13	323	35.3	10		RRLL	137	-	-	ch	cw	6	2	3	D	-	-					
14	321	35.3	9.47	RRLL	RKLL	226-248	-	+	er, pe, ex, g, ch	cw	5	2	3	C	+	+		F	1	SA	+
17	322	35.4	8.89	RRLW	RRLL	197-221	+	+	v, ch, ex, er, g	cw	6	2	3	D	+	+				S3 and 4	+
18	393	43.1	8.39	RKLR		207	+	-	ch, ex, g, n, cy, pl, v, er	cw	5	2	3	D	+	+				S3 and 4	+
19	331	36.4	8.33	RKLL	RKLL	163-187	-	-	cy, n	cw	6	2	3	D	+	+					
22	324	35.8	8.8		RRKL	613	-	-	n, cy, v	cw	6	2	3	D	+	+	2	GE	1	all	+
23	334	36.8	7.06				+	+	ch, n, ex, m, pl, v	cw	6	2	3	D	-	-					
30	291	32	9	RRLW	RRLL	232-258	-	+	v, ch, g, n, pl	cw	5	1	2	D	+	-			4	All	+
31	323	35.6	8.21	RKLK		195	+	+	ch, ex, n, pl, v, er	cw	6	2	3	D	+	+	1			S3 and 4	
32	386	41.2	9.51				-	+	m, ch	cw	1	0	0	A	+	+				S3 and 4	+
36	534	58.9	8.97				+	-	v, ch, n, g	cw	6	2	3	D	+	-				SA	+
37	398	45.2	5.93	RRLL	RRLL	246-283	-	+	ch, n, ex, v, er, g	cw	6	2	3	C	+	+					
38	473	52.2	6.41				+	-	ex, ch, v, n, cy, pl	cw	6	2	3	D	+	-	1	CE,ME	3		
43	297	32.6	9.49	RRML	RKLL	241-273	-	+	er, ch, cy, v, ex	cw	6	2	3	C	+	+					
44	414	46	7.74		RKLL	103	-	-	n, cy	cw	6	2	3	D	+	+		TS	1	all	+
45	318	33.5	8.6		RELL	195	+	+	ch, cy, v, pe	cw	5	2	3	D	+	+		TS	2	S3 and 4	+
46	324	35.8	9.67	RRLL	RRLL	263-292	-	+	n, er, cy, ct, ch, m, v	cw	6	2	3	D	+	+				all	+
47	326	35.6	9.17	RRML	RKLL	241-273	-	+	v, g, ch	cw	6	2	3	C	+	+					
49	525	58.4	9.29				+	-	ex, ch, v, er	cw	6	2	3	D	+	-	2			all	+
50	427	46	9.47				+	-	ch, n	cw	4	1	1	D	+	+	1			SA and S3	+
51	359	39.8	5.21	RRLL		406	-	-	cy, ch, n, pl	cw	2	1	0	C	-	-					
53	322	35.4	9.05	RRLL	RRLL	213-239	-	-	cy, ch	cw	5	2	3	C	+	+		F	5		
54	533	58.9	9.79	RRLL	RRLL	253-281	-	+	n, cy, er	cw, n	6	2	3	D	+	+	1	ES, S, F	3	all	+
56	322	35.6	9.61	RRLL	RKLL	222-240	+	+	ex, er, v, g, ch, n	cw	5	2	3	C	+	+		F	37		
62	334	36	9.94		RRLL	217	+	+	ch, n, ex, v, cy	cw	6	2	3	D	-	-					
63	518	57.6	9.54				+	+	ch, ex, er, n, cy, m	cw	6	2	3	D	-	-					
64	322	35.9	10.32		RKVL	358	-	-	ch, n	cw	6	2	3	D	-	-					
65	319	34.3	9.01		RRLL	187	-	-	n, ch, cy, v	cw	6	2	3	D	-	-					
66	348	38.9	5.87	RRLL	RRML	265-289	-	+	cy, cy_pe, pe, er, pl	cw	5	2	3	C	+	+		F	4		
70	387	42.7	7.71	RKLR		204	+	-	ch, n	cw	6	2	3	D	+	+				all	+
71	529	58.6	5.92				+	-	ch, n, ex, v, m	cw	6	2	3	D	+	+					+
72	407	46.1	5.57	RRLL	RRLL	318-350	-	-	n, cy, ch	cw	6	2	3	C	+	-		F	10		+
73	223	24.6	9.03		RKLL	250	+	-	ch, n	cw	6	2	3	D	+	+	1			all	+
74	526	58.6	9.29				+	-	ch, ex, v, er, g	cw	6	2	3	D	+	-				all	+
75	327	36.1	9.92		GRLL		+	+	ch, v, n	cw	6	2	3	D	-	-					
76	335	36.8	8.76		RRLL	224	+	-	ex, ch, v	cw	6	1	3	D	-	-					
78	400	45.4	6.12	RRLL	RRLL	227-258	+	+	ch, ex, v, g, n	cw	6	2	3	C	+	+					
79	536	59	9.04				+	-	v, ch, n	cw	6	2	3	D	+	+				all	+
80	327	36.1	9.68				+	+	ch, v	cw	6	2	3	D	-	-					
81	327	36	9.13		GRLL		+	-	ex, er, ch, v	cw	6	2	3	D	+	+					
82	334	37.2	9.49	RRLL	REYL	242-253	-	+	cy, cy_n, ch, n	cw	6	2	3	B	-	-					
83	322	35.4	8.95	RRLL	RKLL	237-260	-	+	er, ex, g, ch, cy, v	cw	5	2	3	C	+	-		F	16		
84	547	60.7	5.25				-	+	ch, pl, n	cw	6	2	3	A	+	+					
85	318	33.4	7.84		RKLL	203	+	+	ch, cy, cy_n, v, pe	cw	5	2	3	D	+	+				S3 and 4	
86	330	37	9.52	RRLL	REYL	241-252	-	+	cy, cy_n, n, ch, pl, v	cw	5	2	3	B	-	-					
91	321	35.2	9	RKLL	RRLL	274-301	-	+	n, cy, cy_pe, v, er, ct	cw	6	2	3	B	+	+	1	F	1	all	+
92	331	36.3	7.82		RRLL	267	+	+	pl, er, v, n	cw	6	2	3	D	+	+		S	1	all	+
93	347	37.7	5.16	RRLL	RRFL	246-272	-	+	cy, ex, pe	cw	3	2	3	C	+	+		F	1		+
95	323	34.8	9.47		RRLL	214	+	+	ex, v, ch, g, pl	cw	6	2	3	D	-	-					
96	327	36.6	10.22		RKVL	154	-	-	n, cy, pl, ct_pl, ch, ct	cw	6	2	3	D	-	-					
97	402	45.1	10.48		RRVL	228	+	+	v, g, n, pl, ex	cw	6	2	3	D	-	-					
99	324	36.2	8.24	RRLL	RRML	277-301	-	+	cy, er, ch, pl, v	cw	5	2	3	C	+	-		F	10		

LuPME8, LuPME51, LuPME97, and LuPME22 have two PME domains. **(SP)**: Presence of signal peptide; **(TM)**: Presence of transmembrane domain. **Subcellular localization:** ch: chloroplast; cw: cell wall; cy: cytosol; er: endoplasmic reticulum; ex: extracellular/cell wall; g: Golgi apparatus; m: mitochondria; n: nuclear; pl: plasma membrane; v: vacuolar membrane; pe: peroxisome; ct: cytoskeleton. **(AS)**: Number of conserved residues at active site (out of 6); **(ST)**: Number of conserved stabilizer residues (out of 2). CR: Number of conserved catalytic residues (out of 3). Expression: A gene was reported as positive if the coverage with the EST or assembled RNAseq sequence was higher than 80% and the identity higher than 98%. **(OE)** Overall transcript expression based on all the methods assessed: (+) expression was detected with at least one of the methods; (-) expression not detected in any of the methods. **(FL)** Gene expression based on qRT-PCR (Fluidigm): (+) it was expressed in at least one tissue; (-) no expression; (NA) no assay done. **(UF):** Number of genes aligning with unigenes reported by Fenart *et al.*[35], 80% coverage and 98%identity. **(EV)** Venglat *et al.*[36] ESTs, 80% coverage and 98%identity: (F) flower; (S) stem; (SP) stem peel; (ES) etiolated seedling; (L) leaf; (GE) globular embryo; (HE) heart embryo; (TE) torpedo embryo; (ME) mature embryo; (TS) torpedo seed coat; (GS) globular seed coat; (EP) endosperm pooled. **(EN)** Number of ESTs from NCBI as of April 2013. **(RS)**: Alignment with RNAseq assembled sequences data (80% coverage and 98%identity) obtained from one kp project at different positions in the stem: (SA) shoot apaical meristem; (S3): stem 3; (S4) stem 4. **(RSA)**: RNAseq shoot apical meristem.

**Table 2:** Important features of type-2 LuPMEs

**LuPME**	**Mature protein**			**Subcellular localization**				**# of Conserved residues**			**Tree**	**Expression**						
	**AA**	**Kda**	**pI**	**SP**	**TM**	**WolF PSORT**	**Plant-mPLoc**	**AS**	**ST**	**CR**	**Group**	**OE**	**FL**	**UF**	**EV**	**EN**	**RS**	**RSA**
2	192	21	4.8	+	-	ch, ex, v	cw	1	1	2	E	-	-			0		
4	305	34.4	6.5	-	-	n, cy, m, ch, ex	cw	5	1	3	E	-	-			0		
10	348	38.2	8.4	+	+	ch, ex, v, m	cw	4	2	3	E	+	+		ES, L, GE, HE, TE, ME	12	All	+
12	223	23.1	8.7	-	-	ch, n, m	cw	2	0	2	C	+	-			0		+
15	267	29.9	5.7	-	-	n, cy, pl	cw	3	1	2	E	+	+			0		
16	329	37.1	9.4	+	-	ch	cw	5	1	3	E	+	-			0		+
20	252	27.6	8.9	-	-	cy, ch, ex, n, m	cw	5	1	3	D	-	-			0		
21	352	39.1	8.5	-	+	ex, er, ch, cy, m, v	cw	6	2	3	E	+	+			0	All	+
24	414	45.4	8.3	-	+	ex, ch, v, er	cw	5	2	3	E	+	+	1		0	All	
25	319	34.7	8.5	+	-	ch, ex, m, v	cw	4	1	3	E	+	-			0		+
26	322	35.4	9.5	+	-	ch	cw	5	1	3	E	+	NA			0	all	+
27	363	39.7	8.9	+	+	ch, v, n, pl	cw	6	2	3	E	+	+			0	SA and S4	+
28	74	8.5	11.2	-	-	cy, ch, n, pl	cw, ch	0	0	0	D	+	+			0		
29	356	39.3	9.1	+	+	ch, v, n, pl	cw	6	2	3	E	+	+		F	2	all	+
33	219	24.8	4.9	-	-	cy, ch, n, pe	cw	5	0	2	A	-	-			0		
34	317	35.5	7.7	-	-	cy, n, ch	cw	6	2	3	E	+	+	1		0		+
35	145	15.9	9.1	-	-	n, ch, cy	cw	3	0	1	D	-	-			0		
39	348	38.8	7.8	+	+	v, cy, ch, m	cw	4	2	3	E	+	+			0		
40	316	35.8	9	+	-	ch, n, m	cw	5	1	3	E	+	+		TSC	1		
41	340	37.3	6.3	+	-	ex, v, er, ch, n	cw	3	2	3	E	+	+			0		
42	216	24.4	8.4	-	-	m, ch, v, n	cw, n	1	0	0	E	-	NA			0		
48	379	40.2	8.7	-	-	ch, m, n	cw	3	0	2	C	+	+			0		+
52	336	37.6	6	-	-	cy, er, n	cw	6	1	3	A	+	-			0		+
55	330	36.2	9	+	-	ch, v, ex, n	cw	3	1	3	E	+	+			0		
57	260	28.8	6.8	-	-	n, cy, ct, ex	cw	2	1	3	E	-	-			0		
58	123	14.4	9.4	-	-	cy, n	cw	3	0	1	C	+	-		F	1		
59	318	35.6	7.7	-	-	cy, n, ct, ct_pl	cw	6	2	3	E	+	+			0	SA	+
60	325	35.2	8.4	+	+	ch, ex	cw	4	1	3	E	-	-			0		
61	302	31.9	9.4	-	-	cy, ch, m, n	cw	6	2	3	D	+	+	1		0	S3 and 4	
67	418	45.9	5.6	-	+	v, g, ex, ch	cw	4	2	3	E	+	+			0	All	
68	316	35.1	9.4	-	-	m, ch, n	cw	6	2	3	E	+	+		ES	1	SA	+
69	220	24.5	7.9	-	-	ch, n, cy	cw	5	2	3	D	+	+			0		
77	318	35.2	7.7	+	-	ch, n	cw	5	1	3	E	+	+			0		+
87	348	38.5	7.3	-	-	cy, ct, n	cw	3	2	3	E	-	-			0		
88	140	15.5	8.9	-	-	cy, ch, m	cw	1	0	0	E	-	-			0		
89	311	33.6	5.2	-	-	n, ch, cy, ex	n	3	2	2	E	+	+			0		+
90	339	37.1	6.3	+	-	n, er, er_pl, m, pl, ch, cy	cw	3	2	3	E	+	+			0		+
94	332	37.6	8.5	+	-	ex, v, er, cy	cw	3	1	2	E	+	+			0		
98	354	40.1	8.1	+	-	ch, n, ex, cy	cw	5	1	3	E	-	-			0		
100	289	32.3	7	-	-	cy, n, ct, ch	cw	1	0	2	E	-	NA			0		
101	318	36.2	7.2	+	-	ex, v, er, ch, cy	cw	4	1	2	E	+	+		F	3		
102	317	35.1	5.5	-	-	cy, n, ct	cw	5	2	3	E	+	+			0	All	+
103	359	39.8	8.9	+	-	ch, cy, n, m, ex	cw	6	2	3	E	+	+			0	all	+
104	349	38.4	9.3	-	-	m, ch_m, ch	cw	4	1	3	E	-	-			0		
105	343	37.6	9.1	-	-	ch, cy	cw	6	2	3	E	+	+	1		0	SA	+

LuPME8, LuPME51, LuPME97, and LuPME22 have two PME domains. **(SP)**: Presence of signal peptide; **(TM)**: Presence of transmembrane domain. **Subcellular localization:** ch: chloroplast; cw: cell wall; cy: cytosol; er: endoplasmic reticulum; ex: extracellular/cell wall; g: Golgi apparatus; m: mitochondria; n: nuclear; pl: plasma membrane; v: vacuolar membrane; pe: peroxisome; ct: cytoskeleton. **(AS)**: Number of conserved residues at active site (out of 6); **(ST)**: Number of conserved stabilizer residues (out of 2). CR: Number of conserved catalytic residues (out of 3). Expression: A gene was reported as positive if the coverage with the EST or assembled RNAseq sequence was higher than 80% and the identity higher than 98%. **(OE)** Overall expression based on all the methods assessed: (+) expression was detected with at least one of the methods; (-) expression not detected in any of the methods. **(FL)** Gene expression based on qRT-PCR (Fluidigm): (+) it was expressed in at least one tissue; (-) no expression; (NA) no assay done. **(UF):** Number of genes aligning with unigenes reported by Fenart *et al.*[35], 80% coverage and 98%identity. **(EV)** Venglat *et al.*[36] ESTs, 80% coverage and 98%identity: (F) flower; (S) stem; (SP) stem peel; (ES) etiolated seedling; (L) leaf; (GE) globular embryo; (HE) heart embryo; (TE) torpedo embryo; (ME) mature embryo; (TS) torpedo seed coat; (GS) globular seed coat; (EP) endosperm pooled. **(EN)** Number of ESTs from NCBI as of April 2013. **(RS)**: Alignment with RNAseq assembled sequences data (80% coverage and 98%identity) obtained from the OneKP project at different positions in the stem: (SA) shoot apical meristem; (S3): stem 3; (S4) stem 4. **(RSA)**: RNAseq shoot apical meristem.

We used the same Fluidigm qRT-PCR array system to assay transcription of 94 out of 95 predicted PMEIs. 66/94 genes (70.2%) were detected in one or more tissues (Figure 2B), and 28/94 (29.8%) PMEIs were not detected in any of the tissues. However, 17/29 of the predicted PMEIs that were not detected or assayed by qRT-PCR were identified among either public ESTs collections or in RNAseq data from developing stems (Table 3). Together, these data provide evidence that at least 83/95 (87.4%) of the predicted PMEIs are transcribed.

**Table 3:** Important features of LuPMEIs

**LuPMEI**	**Mature prot.**			**Subcellular localization**				**Conserved residues**							**Expression**						
	**AA**	**Kda**	**pI**	**SP**	**TM**	**WolF PSORT**	**Plant-mPLoc**	*	**§**	**□**	**£2**	**£3**	**¥**	**P55**	**OE**	**FL**	**UF**	**EV**	**EN**	**RS**	**RSA**
1	155	16.1	6.8	+	-	ex, m, v, ch	pl	10	4	4	1	0	4	N	+	+		EP	1		
2	174	18.9	8.6	-	-	ch, cy, m, n	pl, ch	8	2	2	0	2	2	A	-	-			0		
3	162	17.9	6.1	+	-	ex, ch	pl	6	3	2	1	2	4	G	+	+			0		+
4	113	12.6	4.9	-	-	ch, n, m, pl, g_pl	pl, n	2	1	4	1	2	2	-	-	-			0		
5	159	17	4.9	+	-	ex, ch, m	pl	9	5	4	3	1	4	P	+	-		HE	1		
6	177	18.7	8.8	+	+	v, pl, ex	pl	11	5	4	2	1	4	A	+	-		GS	1		
7	155	16.8	5.6	+	+	ex, v, ch, n	pl	11	4	3	1	3	4	A	-	-			0		
8	167	17.8	5.3	-	+	ex, ch, v	pl	7	2	3	2	1	4	T	-	-			0		
9	192	21.5	10	+	+	ch	pl	5	4	4	1	1	4	T	-	-			0		
10	199	21.2	5.1	+	-	ex, ch, m, v	pl	7	2	4	0	0	4	P	-	-			0		
11	296	33.7	4.7	-	-	n	n	6	3	4	1	2	4	H	-	-			0		
12	155	16.7	8.4	+	-	ex, ch	pl	8	4	2	1	0	3	A	+	+		ES	1		+
13	160	16.8	8.3	+	-	ex, ch, v	pl	11	4	2	1	0	4	S	+	-			0	S4	+
14	159	17.4	5.1	+	-	ch, v, ex	pl	11	3	4	2	1	4	S	+	+			0		
15	159	17.3	5.1	+	-	pl, ch, er, ex	pl	11	3	4	2	1	4	S	+	+			0		
16	198	21.3	5.1	+	-	ex	pl	7	5	4	3	1	4	I	+	+		GS	1	SA and S3	+
17	187	20.3	4.5	+	+	ch	pl, n	8	4	3	1	2	4	A	+	+		HE, F	2	S4	+
18	181	19.5	8.3	+	-	ch, m, v	pl	8	3	3	3	1	4	A	+	+		TS	1	SA	+
19	159	17	5	+	-	ex, ch, m	pl	9	5	4	3	1	4	P	+	+		GE, F	4		
20	239	26	5.6	-	+	ch, ch_m, n, m, cy	pl	9	4	4	2	1	4	S	+	-			0	S3 and 4	+
21	161	17.2	7.9	+	-	ch, ex, cy	pl	11	3	4	1	2	4	A	-	-			0		
22	483	55.4	4.6	+	-	ex, v, g, n	n	5	3	4	1	2	4	P	+	+			0		+
23	198	20.8	4.9	+	-	ch, ex, cy	pl	6	3	3	1	1	4	P	+	+		F	5		
24	180	19.6	8.9	+	-	v, ch, ex	pl	9	3	3	0	0	4	P	+	+		F	3		
25	172	18.6	4.9	+	-	ex, ch	pl	11	5	3	3	1	4	K	+	+			0		
26	154	16.2	5.5	+	-	ex, m, ch	pl	10	4	2	1	1	4	A	+	+		L	1	SA and S3	+
27	149	16.4	4.7	+	-	ex, v, ch	pl	10	3	4	1	2	4	N	+	+			0		
28	166	17.1	8.3	+	-	ex, pl, ch, m, v	pl	11	4	2	1	0	4	S	+	+			0	SA	+
29	149	16.7	4.8	+	-	v, ch, ex, er	pl	10	2	4	1	1	4	N	+	+		EP, HE	3		
30	149	16.4	4.6	+	+	ex, v, cy, m	pl	10	2	4	1	0	4	T	+	+		EP,GS	24		
31	165	17.8	5.5	+	-	pl, g, ex, er	pl	11	4	2	1	1	3	K	-	-			0		
32	155	16.5	8.4	+	+	ch, v, cy, n, m	pl	10	4	3	1	0	4	T	+	+			0		
33	152	15.9	5.1	+	-	ex, ch	pl	9	4	3	1	2	4	A	+	-		GS	1		
34	152	16.2	4.7	+	+	v, pl	pl	11	4	4	2	2	4	A	+	+			0		
35	154	16.1	4.6	+	-	ex, ch, n	pl	10	4	4	2	2	4	A	+	-			0		+
36	152	16.3	4.7	+	-	ex, ch, cy, m	pl	11	5	4	3	2	4	A	+	+			0		
37	167	17.9	5.4	-	-	ch, cy, n, ct_n	pl	8	4	4	2	1	4	P	+	+			0		+
38	163	17.8	7.8	+	-	ex, ch, pl	pl	9	5	3	1	2	4	P	+	+			0	S3	+
39	158	17.4	4.5	-	-	ch, ex, n	pl	10	5	2	3	1	4	K	+	-			0		+
40	164	17.6	5.7	+	+	v, ex, pl, m, er_pl, n	pl	9	5	3	2	2	4	A	+	+	1	HE	1		+
41	151	16.1	5.3	+	-	ch, v, n, m, ex	pl	11	5	2	1	2	4	M	+	+		HE, GS	2		
42	159	17.2	6.1	+	-	ex, ch, v, m	pl	10	3	4	1	1	4	S	+	+			0		
43	181	19.6	9.5	+	-	ch, v	pl	7	4	4	1	1	4	I	+	+			0	SA and S3	+
44	187	19.9	6.8	+	+	ex, v	pl	7	5	4	2	1	4	I	+	+			0	All	+
45	159	17.3	6.5	+	-	ex, ch, n, cy	pl	7	3	4	3	1	4	I	+	+			0	SA	+
46	389	45	5.1	-	-	n	n	5	3	4	1	2	4	P	+	+			0		+
47	188	20.2	8.4	+	-	ex, v, ch, m	pl, n	7	3	4	0	1	4	P	+	+			0		
48	193	21.2	7.7	+	+	n, ex, ch, cy, v	pl, n	7	3	4	1	0	4	P	+	+		F	2		
49	197	21.5	6.9	+	+	ex, ch, n, m, v	pl, n	8	3	4	1	0	4	P	-	NA			0		
50	467	50.6	6.5	-	-	n, ch, pl, m	pl, n	7	4	3	1	3	4	A	+	+			0		
51	368	37.4	4.3	+	+	ex, ch, v, n	n	8	4	2	1	2	4	P	+	+			0		+
52	65	7.3	4.5	-	-	cy, n, ch, pl, ex	pl, n	5	1	1	0	1	1	-	+	+			0		
53	161	17.8	9	+	+	ch, ex	pl	7	5	4	3	1	4	I	+	-		S	1		+
54	175	18.7	6.1	+	-	ch, m, ex	pl	7	5	4	3	1	4	I	+	+			0	All	+
55	141	15.3	5.9	-	-	ch, m	cw	4	1	4	2	1	3	-	+	+			0		
56	167	18.8	8.3	+	-	ch, ex, cy, m, pl	pl, n	7	4	3	1	2	4	D	+	+			0		+
57	154	16	6.4	+	+	ex, ch, cy, m	pl	9	4	4	2	0	4	N	+	+		GE	1		
58	195	21.1	5.2	+	-	ex, ch	pl	7	5	4	3	1	4	I	+	-			0	all	
59	173	18.9	4.5	+	-	ex, ch, n	pl	10	5	2	3	1	4	K	+	+			0		
60	195	21.7	9.9	+	-	ch	pl	6	4	4	1	1	4	T	+	-			0		+
61	468	51.7	8.7	-	-	n, ch, cy	pl, n	7	4	3	3	1	3	G	+	-			0	All	+
62	118	13.2	6.9	-	+	ch, cy, n, ex	pl	4	1	3	2	3	2	-	-	-			0		
63	170	18.3	5.9	+	-	ch, v, ex, m	pl	7	5	4	2	2	4	V	+	+		TE	1		+
64	177	19.1	10	+	-	ch	pl	7	4	4	3	1	4	I	+	+			0	SA	+
65	180	19.1	5.3	+	-	ch, m	pl	6	5	4	3	1	4	I	+	+	1		0	all	+
66	160	17.8	7.8	+	-	ex, ch, n, cy	pl	7	3	4	0	2	4	T	+	+		TS, EP	4	SA	
67	174	18.8	4.6	+	-	ex, ch	pl	11	5	4	3	1	4	K	+	+			0		
68	177	19	5.5	+	-	ch, ex, v, m	pl	7	5	4	3	2	4	V	+	+			0		+
69	179	19.3	9.2	+	-	ch	pl	7	5	4	3	2	4	I	+	-			0		+
70	177	19.2	10.1	+	-	ch	pl	7	4	4	3	1	4	I	+	-			0	All	+
71	183	19.6	5.7	+	-	ch, m	pl	6	5	4	3	1	4	I	+	+			0		+
72	183	20	9.4	+	-	ch, n	pl	7	5	4	1	1	4	I	+	+			0		+
73	212	22.6	7.7	+	-	ex, m, v	pl	7	5	4	2	1	4	I	+	+			0	SA and S4	+
74	159	17.4	5.5	+	-	ch, ex, n, cy, m	pl	6	3	4	3	1	3	I	+	+			0	S3 and 4	+
75	170	19.1	9	+	-	ch, pl	pl, n	7	5	3	1	2	4	D	+	+			0	S4	+
76	228	25	9	+	-	ch, v, ex	n	9	3	2	0	0	4	P	+	+		F	1		
77	220	23	4.6	-	-	ch, n	pl	9	3	3	1	1	4	P	+	+			0		
78	163	17.7	5.7	+	-	pl, g, ex, ch	pl	11	4	2	1	1	4	K	+	-			0	SA and S3	
79	155	16.6	6.9	+	+	er, ex, er_pl, ch, m, pl, cy	pl	10	4	3	1	0	4	T	+	-			0	S3 and 4	
80	229	24.9	5.7	+	-	ch, ex	pl, n	7	4	3	2	2	4	A	-	-			0		
81	184	19.3	4.8	-	-	ch, m, n	pl	5	2	4	2	1	3	P	+	-	1		0	S3	+
82	334	34.4	4.4	+	+	ex, ch, v, n	n	8	4	2	1	2	4	P	+	+			0		
83	249	26.9	4.7	+	-	ch, ex, v	pl, n	7	4	3	1	2	4	A	+	+			0		
84	193	20.5	4.7	+	-	ex, ch, v	pl	7	5	4	2	1	4	V	+	+			0	S3	+
85	156	16.9	8.4	+	-	ex, v, pl	pl	8	3	3	0	0	4	R	+	+			0	All	+
86	150	16.2	9.2	+	+	v, ex, pl	pl	10	2	4	0	1	4	A	+	+			0	All	+
87	162	17.7	9	+	-	ch	pl	7	5	4	3	1	4	I	+	+			0		+
88	172	18.3	5.4	+	+	ch	pl	7	5	4	3	1	4	I	+	+		TS	1	All	+
89	155	16.6	8.8	+	+	ex, pl, v, ch, cy, m	pl	10	2	4	0	1	4	A	+	+			0	S3 and 4	+
90	188	19.9	5.7	-	-	ch, m, cy	pl, cy	6	3	3	2	1	2	P	+	+		F	1		+
91	327	36.6	9.2	-	+	pl, ch, m, er	pl	9	4	3	2	3	4	T	+	+			0		
92	162	17.9	7.8	+	-	ex, ch	pl	6	3	2	1	2	4	G	+	+			0	all	+
93	156	16.5	4.4	+	+	n, ex, m, er_pl, pl, er	pl	11	5	4	2	2	4	G	+	-		EP	1		
94	331	35.9	6.3	-	+	n, g, pl, er_pl, cy	cy	9	4	4	2	1	4	S	+	+	1	TE	1	SA and S4	+
95	157	17.3	5.3	-	-	cy, ch, n, ex	pl	8	4	3	1	2	3	P	+	+			0		+

**(SP)**: presence of signal peptide; **(TM)**: presence of transmembrane domain. **Subcellular localization:** ch: chloroplast; cw: cell wall; cy: cytosol; er: endoplasmic reticulum; ex: extracellular/cell wall; g: Golgi apparatus; m: mitochondria; n: nuclear; pl: plasma membrane; v: vacuolar membrane; pe: peroxisome; ct: cytoskeleton. Number of conserved residues at each one of the following: (*) non polar bundle-hairpin interface (out of 12); **(§)** polar bundle-hairpin interface (out of 5); **(□)** Polar interacting with PME aromatic residues (out of 5); **(£2)** acidic patch on α2 helix (out of 3); **(£3)** acidic patch on α3 helix (out of 3); **(¥)** disulphide bridge (out of 4). Expression: A gene was reported as positive if the coverage with the EST or assembled RNAseq sequence was higher than 80% and the identity higher than 98%. **(OE)** Overall expression based on all the methods assessed: (+) expression was detected with at least one of the methods; (-) expression not detected in any of the methods. **(FL)** Gene expression based on qRT-PCR (Fluidigm): (+) it was expressed in at least one tissue; (-) no expression; (NA) no assay done. **(UF):** Number of genes aligning with unigenes reported by Fenart *et al.*[35], 80% coverage and 98% identity. **(EV)** Venglat *et al.*[36] ESTs, 80% coverage and 98%identity: (F) flower; (S) stem; (SP) stem peel; (ES) etiolated seedling; (L) leaf; (GE) globular embryo; (HE) heart embryo; (TE) torpedo embryo; (ME) mature embryo; (TS) torpedo seed coat; (GS) globular seed coat; (EP) endosperm pooled. **(EN)** Number of ESTs from NCBI as of April 2013. **(RS)**: Alignment with RNAseq assembled sequences data (80% coverage and 98%identity) obtained from one kp project at different positions in the stem: (SA) shoot apical meristem; (S3): stem 3; (S4) stem 4. **(RSA)**: RNAseq shoot apical meristem.

Using qRT-PCR and by querying previously published and unpublished transcript databases, we were able to confirm that 77/105 and 83/95 of the predicted LuPMEs and LuPMEIs, respectively, are transcribed. The remaining genes might also be transcribed but under conditions different from those assayed to date. We note, for example, that none of the tissues surveyed to date are from plants subjected to stress, which is likely to induce PMEs that may not be otherwise transcribed. Among genes that are known to be transcribed, we found transcripts expressed in fibers and fiber bearing tissues, during either elongation (7 PMEs, 3 PMEIs), thickening (16 PMEs, 10 PMEIs), or maturation and thickening (19 PMEs, 24 PMEIs) (Table 4). These genes are primary targets for manipulation by reverse genetics, in order to develop flax feedstocks with modified fiber properties.

**Table 4:** Genes putatively associated with fiber development during elongation, thickening, and maturation processes

	**Gene**	**Expression pattern**	**Homologous to**
§	LuPME1	L-E	AtPME35
§	LuPME3	O-X; L-E	PttPME1
§	LuPME5	L-E	
¥	LuPME7	O-X; E-L	PttPME1
¥	LuPME8	E-L	
§	LuPME9	L-E	
¥	LuPME10	O-X; E-L; EC-LC	
¥	LuPME17	O-X; E-L; EC-LC	
¥	LuPME18	O-X; E-L	
*	LuPME21	SA-S	
§	LuPME24	L-E	
¥	LuPME27	O-X; EC-LC	
§	LuPME31	O-X; L-E	
§	LuPME32	OV	
¥	LuPME34 and LuPME59	E-L	
¥	LuPME39	O-X; E-L	
§	LuPME40	O-X; L-E	
¥	LuPME43	O-X; EC-LC	
¥	LuPME44	O-X; E-L	
*	LuPME46	SA-S	
§	LuPME48	O-X; L-E	
¥	LuPME50 and LuPME73	E-L	
§	LuPME53	L-E	
§	LuPME54	O-X; L-E	
¥	LuPME55	EC-LC	
§	LuPME56	O-X; L-E	
§	LuPME61	O-X; LC-EC	AtPME35
§	LuPME66	L-E; LC-EC	
*	LuPME67	SA-S	
§	LuPME68	O-X; L-E	
§	LuPME69	O-X	
*	LuPME70	SA-S	
§	LuPME71	OV; O-X; LC-EC; LCP	
¥	LuPME73	O-X; EC-LC	
*	LuPME79	SA-S	
§	LuPME81	O-X	
¥	LuPME85	O-X; EC-LC	
§	LuPME91	L-E	
*	LuPME92	SA-S	PttPME1
*	LuPME102	OV; SA-S	
¥	LuPME103	O-X; EC-LC	
¥	LuPME105	O-X; EC-LC	
§	LuPMEI1	O-X; LC-EC	
§	LuPMEI17	LC-EC	
§	LuPMEI19	LC-EC	
§	LuPMEI23	O-X	
§	LuPMEI24	LC-EC	
§	LuPMEI25	LC-EC	
*	LuPMEI27	SA-S	
§	LuPMEI28	LC-EC	
¥	LuPMEI29	EC-LC	
§	LuPMEI38	L-E	
§	LuPMEI40	O-X	
§	LuPMEI41	O-X; L-E; LC-EC	
¥	LuPMEI43	EC-LC	
¥	LuPMEI44	E-L	
*	LuPMEI45	SA-S	
§	LuPMEI51	L-E; LC-EC	
¥	LuPMEI54	O-X; EC-LC	
§	LuPMEI55	OV	
§	LuPMEI56	O-X; L-E	
*	LuPMEI59	SA-S	
§	LuPMEI63 and LuPMEI68	O-X; L-E	
¥	LuPMEI65	O-X; E-L; EC-LC	
¥	LuPMEI66	E-L	
§	LuPMEI68	O-X	
§	LuPMEI71	O-X	
¥	LuPMEI72	EC-LC	
§	LuPMEI74	L-E	
¥	LuPMEI76	EC-LC	
§	LuPMEI82	L-E	
§	LuPMEI83	OV	
¥	LuPMEI85	O-X; E-L	
§	LuPMEI86	L-E	
§	LuPMEI87	O-X	
¥	LuPMEI89	E-L	
§	LuPMEI90	O-X; L-E; LC-EC	
§	LuPMEI94	L-E	
§	LuPMEI95	L-E	

Genes putatively associated with specific fiber development processes are marked by symbols: elongation (*), thickening (¥), and thickening and maturation (§). If two genes are shown in one line it means the primers used amplified both genes. (OV) expressed only in vascular tissues; (SA-S) present in SA but not in the rest of the stem (EF, LF, ECP, LCP); (O-X) present in outer tissues (cortical peel and fibers) but not xylem; (L-E) present in LF but not EF; (E-L) present in EF but not LF; (EC-LC) present in ECP but not LCP; (LC-EC) present in LCP but not ECP; (SA) only present in SA; (LCP) only present in LCP. The genes underlined were found to be “SA-S” using the qRT-PCR data, however, according to RNAseq assembled reads they are present in other parts of the stem in addition to SA. As they were only detected in SA with qRT-PCR it is expected that their expression is higher in the SA.

### Transcript expression patterns

**Figure 3:**
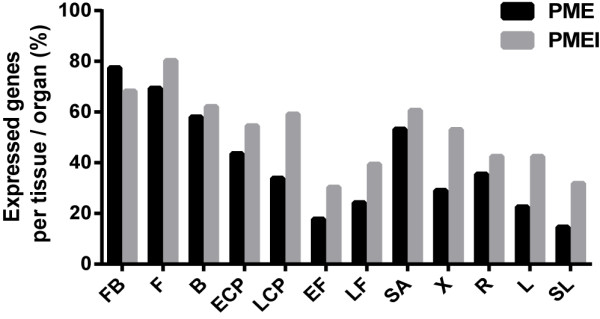
**Percentage of genes detected by qRT-PCR per tissue.** The percentage was calculated based on those genes that showed transcript expression in at least one tissue.

The tissue in which the largest number of expressed PMEs (48/62; 77.4%) was detected was the flower bud. Conversely, the tissue in which the lowest number of PMEs was detected was senescent leaves (9/62; 14.5%). The highest number of PMEIs detected was also in a reproductive tissue (flowers; 53/66 (80.3%). Conversely, the tissue with the fewest detectable PMEIs was early fibers, with only 20/66 (30.3%) (Figure 3).

**Figure 4:**
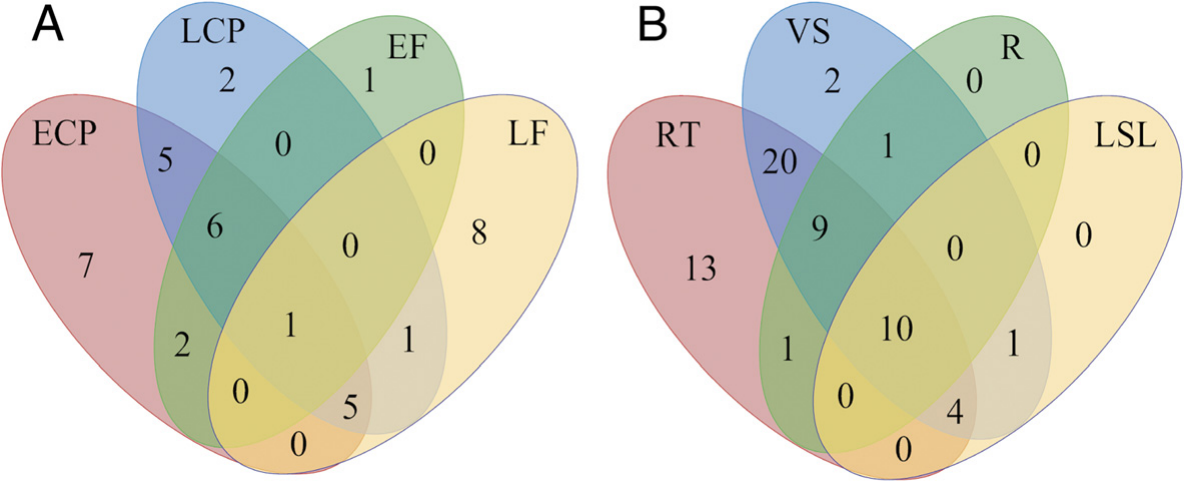
**LuPME transcript expression in various tissues.** Venn diagram showing the number of LuPMEs detected in phloem-fiber containing tissues **(A)** and in tissue systems **(B)** ECP: early cortical peels. LCP: late cortical peels. EF: early fibers. LF: late fibers. RT: reproductive tissues. VS: Vascular tissues at shoot. R: Root . LSL: Leaves and senescent leaves.

**Figure 5:**
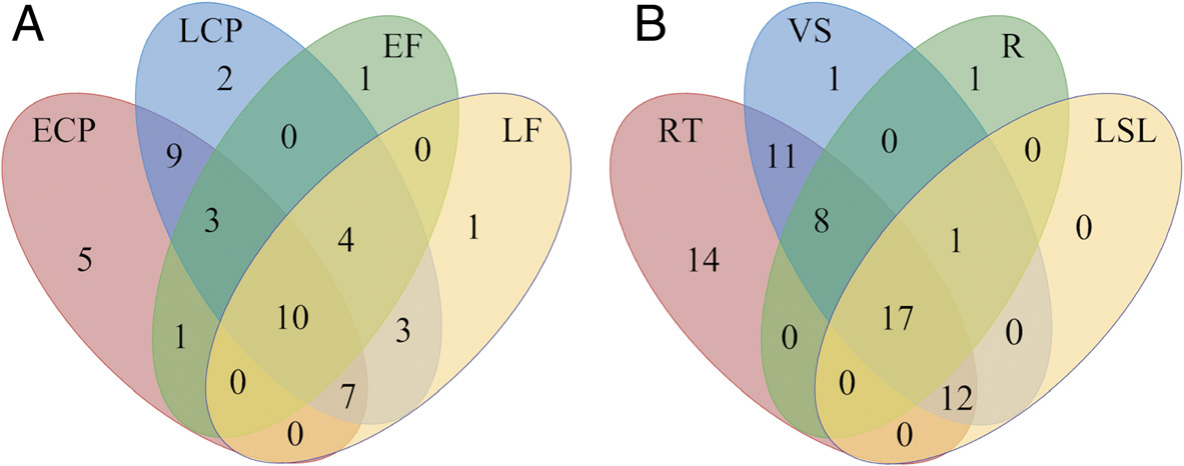
**LuPMEI transcript expression in various tissues.** Venn diagram showing the number of LuPMEIs detected in phloem-fiber containing tissues **(A)** and in tissue systems **(B)** ECP: early cortical peels. LCP: late cortical peels. EF: early fibers. LF: late fibers. RT: reproductive tissues. VS: Vascular tissues at shoot. R: Root . LSL: Leaves and senescent leaves.

We also identified PMEs and PMEIs whose transcript abundance was correlated with phloem fiber development. The transcript expression of 11 PMEs and 20 PMEIs was detected in EF, while 15 PMEs and 26 PMEIs were expressed in LF (Figure 2). 15 PMEI were expressed in both EF and LF while only one PME was expressed in both of these stages. Nine PMEs and five PMEIs were detected in EF but not LF, and conversely 13 PME and 12 PMEI were detected in LF and not EF. In general there were more PMEIs expressed in the fibers. Specifically there were more PMEIs expressed in the LF than in the EF, which might indicate that the inhibitory activity of the PMEIs is low at early stages of fiber development (i.e. EF stage), when fibers actively synthesize secondary cell walls, and demethylesterification of the newly synthesized homogalacturonan is required. However, when the cell wall deposition ceases, in the late fiber stage, PMEIs are expressed, and so the PME activity diminishes. Seven PMEs and three PMEI were expressed in the shoot apex (SA), but not in any other of the stem vascular tissues. Moreover, nine PMEs and six PMEIs were expressed in the early cortical (ECP) peel, but not the late cortical peel (LCP), and three PMEs and nine PMEIs were expressed in late cortical peel but not early cortical peel (Figures 4A and 5A). 13 PMEs and 14 PMEIs were found only in reproductive tissues; and three PMEs and two PMEI were found only in vascular tissues (Figures 4B and 5B). Seven PMEs and six PMEIs showed specific transcript expression in only one tissue/organ; these transcripts were detected in flower buds (four PMEs and two PMEIs), flowers (one PMEI), bolls (one of each), xylem (one PMEI), roots (one PMEI), late cortical peel (one PME), and shoot apex (one PME). Two of these might be important for phloem fiber development: LuPME71, which was detected only in LCP, a fiber containing tissue where secondary cell wall deposition and maturation is taking place, and LuPME102, only detected in the SA, where intrusive growth takes place (Figure 2A-B).

Previous publications have reported transcript expression patterns of specific LuPMEs. Al-Qsous *et al.*[13] found that the transcript abundance of LuPME5 is higher than either LuPME1 or LuPME3 in hypocotyls. Our results were consistent with these observations: The calculated delta-C_T_ of LuPME5 was higher than LuPME1 or LuPME3 in all the tissues tested (Figure 2). Our transcript abundance data also showed that LuPME5 was expressed in the shoot apex, while LuPME3 was not, which could be correlated with the findings that showed that LuPME5 transcript abundance was higher in the upper parts of the hypocotyl after two days of growth, while LuPME3 was higher in the bottom of the hypocotyl [13]. Mareck *et al.*[14] found a very high transcript abundance of LuPME3 in roots, as observed with the promoter fusion in tobacco [11], in which a GUS construct using LuPME3 promoter was used to detect its expression in stems, roots and leaves. The expression was observed in the vascular tissues of roots, shoots and young leaves. This correlates with our results, as we detected LuPME3 transcript expression only in roots, late fibers and the boll. Our study used mature leaves, rather than young leaves, which may explain why we failed to detect transcript expression in this tissue, in contrast to Mareck *et al.*

### Protein subcellular localization

**Figure 6:**
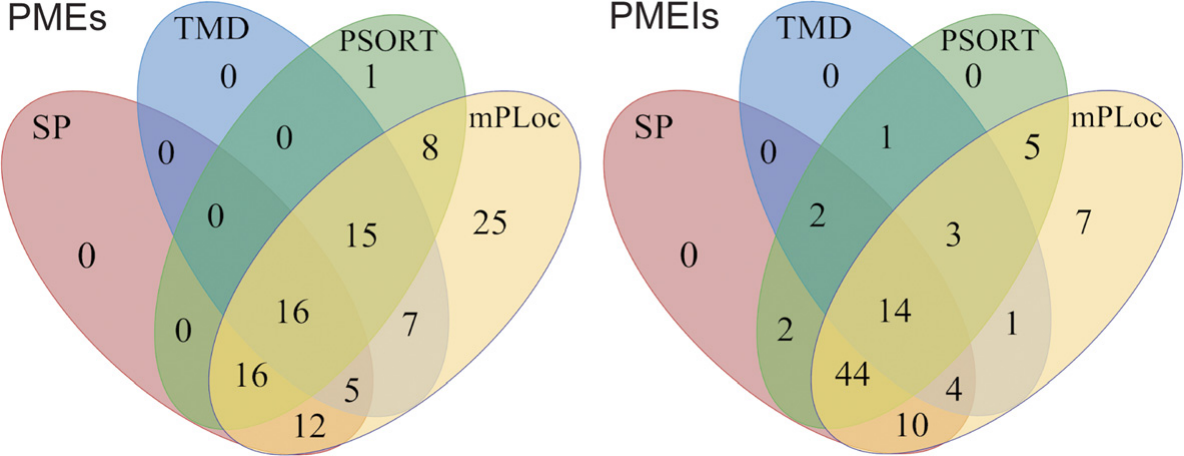
**Subcellular localization of PMEs and PMEIs.** Number of PMEs and PMEIs with transmembrane domains, signal peptides, and predicted to be secreted to the cell wall or plasma membrane using WolF PSORT and Plant-mPLoc.

To be secreted to the cell wall via the Golgi apparatus and secretory pathway, PMEs and PMEIs require an N-terminal signal peptide or a transmembrane domain [4]. As shown in Figure 6, we found that 71/105 LuPMEs had a predicted transmembrane domain and/or signal peptide, and 81/95 LuPMEIs had a transmembrane domain and/or signal peptide (Tables 1, 2 and 3). To further investigate subcellular localization, we used WoLF PSORT and Plant-mPLoc [23,24]. Using Wolf PSORT we found 56/105 LuPMEs, and 71/95 LuPMEIs that were predicted to be secreted to either the cell wall or to the plasma membrane. Plant-mPLoc predicted 104/105 LuPMEs to be secreted to the cell wall, and 88/95 LuPMEIs were predicted to be secreted to the plasma membrane or the cell wall. In total all the LuPMEs and 93/95 LuPMEIs were predicted to be extracellular, based on protein subcellular localization software prediction and/or the presence of signal peptide and/or transmembrane domain. The two LuPMEIs, LuPMEI11 and LuPMEI46 that were not predicted to be extracellular were predicted by both software tools to be localized to the nucleus.

### Cleavage site

**Figure 7:**
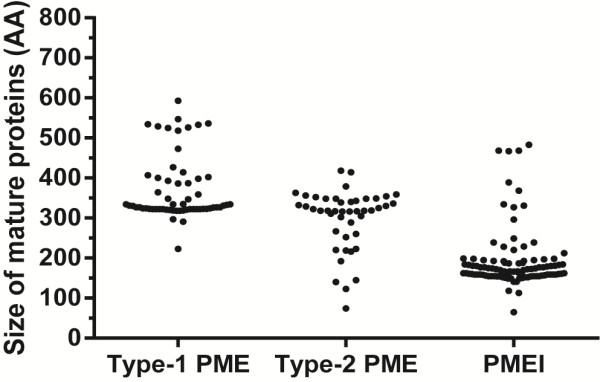
**Size of mature proteins.** The signal peptide and/or cleavage site, if present, were removed.

During their maturation, most Type-1 PMEs are proteolytically cleaved at one of two possible sites between the PMEI domain and the PME domain, before exiting the Golgi apparatus. These sites are designated binding motif 1 (BM1), and binding motif 2 (BM2) (Figure 1), and are separated by between 11–32 amino acids in Arabidopsis [8]. A conserved cleavage site consisting of four residues with the pattern [RKQ][RKEHLN][LDMI][LMAKR] was previously defined by analysis of *A. thaliana* PMEs [4,8]. We identified this pattern at a single site in each of 25/60 of the predicted flax Type-1 PMEs. Moreover, 19/60 flax Type-1 PMEs had two sites that matched the previously defined pattern, and these were separated by between 14 and 33 residues. In 3/60 of the LuPMEs, a novel tetrapeptide motif (RRKL or GRLL) was found in the place of the conserved pattern in BM2. Other novel motifs were also found, but these were all accompanied by a conserved motif in the other binding site, we found RKVA and RRLW in BM1, and REYL and RRFL in BM2. In 13/60 Type-1 PMEs, a cleavage site motif was not found (Table 1). Figure 7 shows the distribution of sizes of the mature proteins of PMEs and PMEIs (after signal peptide and/or pro-region removal, if present).

There was no significant difference (Fisher’s Exact Test p > 0.05) in the distribution of the cleavage site features between the 45/60 predicted PMEs for which evidence of transcription has been found, and the 15/60 predicted PMEs for which no evidence has been found. The inability to cleave out the PMEI domain (pro-region) would presumably prevent the export of the PMEs to the cell wall, according to Wolf *et al.*[8], who showed that unprocessed Type-1PMEs are retained in the Golgi apparatus. Nevertheless, LuPME5 was detected in both the unprocessed and processed forms in cell walls of flax cell cultures [13], and LuPME3 was only detected in the unprocessed form in flax seedlings and callus [14]. This raises the possibility that the processing of Type-1 LuPMEs might be dispensable for the proper functioning or at least localization of the protein.

### Isoelectric point

**Figure 8:**
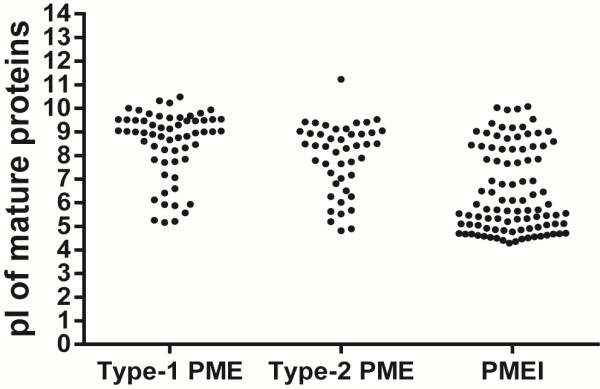
**Isoelectric point of mature proteins.** The signal peptide and/or cleavage site, if present, were removed.

The isoelectric point is one of the factors that influences the action of PMEs (i.e. random, acidic pI, or blockwise, alkaline pI [37]) and so can facilitate either stiffening or loosening of the cell wall. Consequently, the prediction of the pI of the proteins contributes to the definition of their physiological role in the plant. We calculated the pI for the mature proteins (i.e. with any signal peptide or pro-region removed) (Figure 8). Most of the PMEIs (51) had an acidic pI, while 26 had a basic pI (pH $\bar{x}$: 6.48, σ_x_:1.77.). On the other hand, most of the PMEs (70) had a basic (above pH8.0) pI (pH $\bar{x}$: 8.26, σ_x_: 1.46.), while just a few (13) had an acidic pI, below 6: Out of these, four showed tissue specificity: Two in FB: LuPME93 and LuPME37, and two in fiber containing tissues: LCP (LuPME71), and SA (LuPME102). As an acidic pI would lead to random demethylesterification, which ultimately could lead to cell wall loosening; it could be expected that when LuPME71 is expressed in the LCP, it decreases the rigidity of the connections between fibers, while LuPME102 might enhance fiber growth as it could loosen the connections between parenchyma cells to facilitate fiber intrusive growth. Only one of the genes whose transcript expression was detected in the EF or LF had an alkaline pI, this was LuPME66 (pI 5.87), which was expressed in reproductive tissues, xylem, LCP and LF. This could have a role similar to the described for LuPME71.

The wide range of predicted isoelectric points for the mature PMEs (pH 4.75 to 11.25; Tables 1 and 2) is consistent with previous reports from flax. Gaffe *et al*. [38] tested the PME activity from cell wall of flax calli; they found isoforms at pI 5.5, 7, 7.3, 7.8, 8.8, and 10. Mareck *et al.*[39] found a similar range of isoforms in flax calli; they found PMEs with pIs 4.3, 4.8, 6.3, 7.1, 7.6, and 9.6. Alexandre *et al.*[40] found 2 PMEs in hypocotyls, at pH 8 to 9, and at pH 9.5 to 10. Al-Qsous *et al.*[13] observed PME activity in the hypocotyls at 5 different isoelectric points, from pH 7.0 to 10.0. Finally, Mareck *et al.*[14] also found a similar pattern of the isoforms in epicotyls, cotyledons, hypocotyls and roots; they found two neutral, four basic and one strongly basic PME isoform.

### Phylogenetic analysis

**Figure 9:**
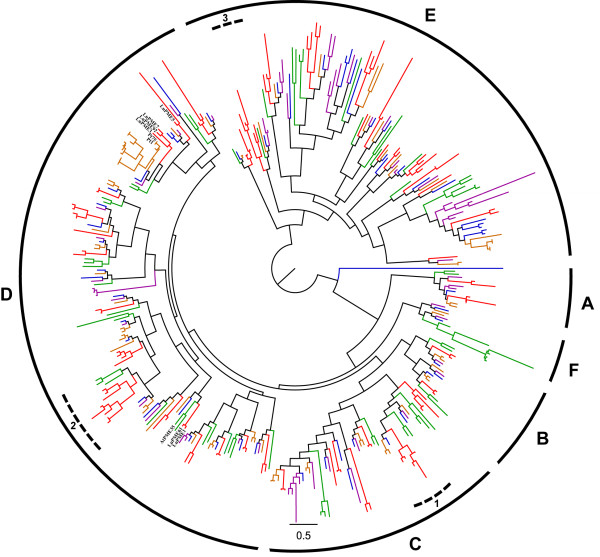
**Maximum likelihood dendrogram of PMEs in flax and related species.** The main groups (labeled **A-F**) and some subgroups are shown. The previously reported LuPMEs and the homologous LuPMEs to PttPME1 and AtPME35 are labeled. Red: Linum usitatissimum; Purple: Manihot esculenta; Blue: Ricinus communis; Orange: Populus trichocarpa; Green: Arabidopsis thaliana. 100 bootstraps and 2 search-replicates (bootstrap values shown in Additional file 4: Figure S2).

**Figure 10:**
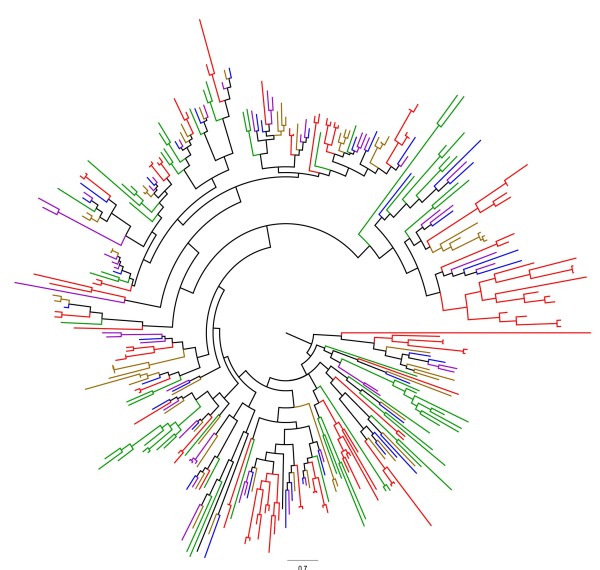
**Maximum likelihood dendrogram of PMEIs in flax and related species.** Red: *Linum usitatissimum*; Purple: *Manihot esculenta;* Blue: *Ricinus communis;* Orange: *Populus trichocarpa;* Green: *Arabidopsis thaliana*. 100 bootstraps and 2 search-replicates (bootstrap values shown in Additional file 5: Figure S3).

To classify the predicted LuPMEs and LuPMEIs on the basis of amino acid sequence similarity and inferred evolutionary relationships, we aligned their amino acid sequences with predicted PMEs and PMEIs from four other angiosperms: cassava (*Manihot esculenta*)*,* castor (*Ricinus communis*)*,* poplar (*Populus trichocarpa*)*,* and *A. thaliana.* These species were chosen because Arabidopsis is a well-characterized model organism, and castor, cassava, and poplar are in the same taxonomic order (Malpighiales) as flax, and whole-genome assemblies are available for each of these species. Following alignment, maximum likelihood phylogenetic trees for PMEs (Figure 9) and PMEIs (Figure 10) were constructed. Based on the groups defined by Louvet *et al.*[41] for Arabidopsis PMEs, the branch length, and the bootstrap values (Additional file 4: Figure S2), six major monophyletic groups of PME could be defined (A, B, C, D, E, and F which correspond to groups 3, 1, 2, 1, 4, and 1 respectively in Louvet *et al.*[41]). Group A included five LuPMEs; three of them were Type-1 PME, and none of them had a cleavage recognition site. The PMEs in all the organisms of group B were Type-1 PMEs, and all the LuPMEs in this group had a cleavage recognition site, and a transmembrane domain, but no predicted signal peptide. Group C was composed of Type-1 and Type-2 PMEs. All of the Type-1 PMEs had a cleavage recognition site, and none of the Type-2 PMEs had either a signal peptide or transmembrane domain. Group D contained the previously described LuPME1, LuPME3, and LuPME5, we did not find any characteristic defining this group based on the parameters we described above (e.g. Table 1). The PMEs of all the species in group E were Type-2 PMEs. Finally group F contained PMEs of all the species analyzed except flax.

In the PMEI phylogenetic tree (Figure 10), groups were distinguished by very low bootstrap values in the base nodes (Additional file 5: Figure S3), making sub-classification of PMEIs ambiguous. Furthermore, we did not find any common sequence features that distinguished subgroups of PMEIs from each other.

Phylogenetic analysis of LuPMEs and LuPMEIs grouped 43 pairs of LuPMEs (out of 105 genes in total) and 39 pairs of LuPMEIs (out of 95 genes in total) at the terminal nodes of the tree (Figures 9 and 10). The remaining genes, 19 PMEs and 17 PMEIs, did not have obvious paralogs. The pairs of genes were confirmed by reciprocal BLASTn and BLASTp to test if they were the best BLAST match. 38/43 pair of LuPMEs and 38/39 pairs of LuPMEIs were found to be the best match to one another. This suggests these probably originated from a recent whole genome duplication event believed to have occurred 5 to 9 MYA [15]. Indeed, for the LuPMEIs, the estimated time of divergence of presumptive paralogs was calculated to be 4.5-8.4 MYA, and for the LuPMEs estimate was 6.4 -11.9 MYA (Additional file 6: Table S2). Lineage-specific expansion of groups of PMEs or PMEIs may indicate that selection had occurred for particular functions in flax. Expansion of at least three sub-groups (C1, D2, and E3) of PMEs was evident in the ML tree (Figure 9).

We identified PMEs genes that have been associated with stem development in previous studies in other species, and found their presumptive homologs in flax. Siedlecka *et al.*[42] found that when the transcript abundance of PttPME1 (accession no. AJ277547) in hybrid Aspen (*Populus tremula* × *tremuloides*) increased, the fiber elongation decreased, and conversely, when the transcript abundance of the gene was low it stimulated fiber elongation. The closest PMEs in *P. trichocarpa* for PttPME1 are Pt1 (POPTR_0001s16250.1) and Pt75 (POPTR_0214s00200.1). Based on the phylogenetic tree, we found three LuPMEs that were closely related to Pt1and Pt75; they were LuPME7, LuPME92, and LuPME3 (Figure 9; Table 1), which were all type-1 PME, as PttPME1. The study of these genes will be important in future studies as they may regulate fiber elongation the same way as in poplar, as the fibers of both plants elongate intrusively. Hongo *et al.*[43] found that the type-1 PME AtPME35 (At3g59010), has a role in strengthening the inflorescence stem of Arabidopsis, by mediating the demethylesterification of the primary cell wall of cortical cells and interfascicular fibers, this gene was suggested to have a blockwise demethylesterification action. We inferred that the common ancestor of LuPME61 and LuPME1 is the likely ortholog to AtPME35 (Figure 9; Table 4). Both LuPME61 and LuPME1 have basic pIs (9.42 and 8.82, respectively), similar to the pI of the mature protein of AtPME35 (pI 8.70), so it is possible that they also have a blockwise demethylesterification activity similar to AtPME35, which leads to stiffening of the cell wall. The study of loss-of-function mutants for these genes in flax might identify informative phenotypes related to stem development.

### Conserved residues in PMEs

**Figure 11:**
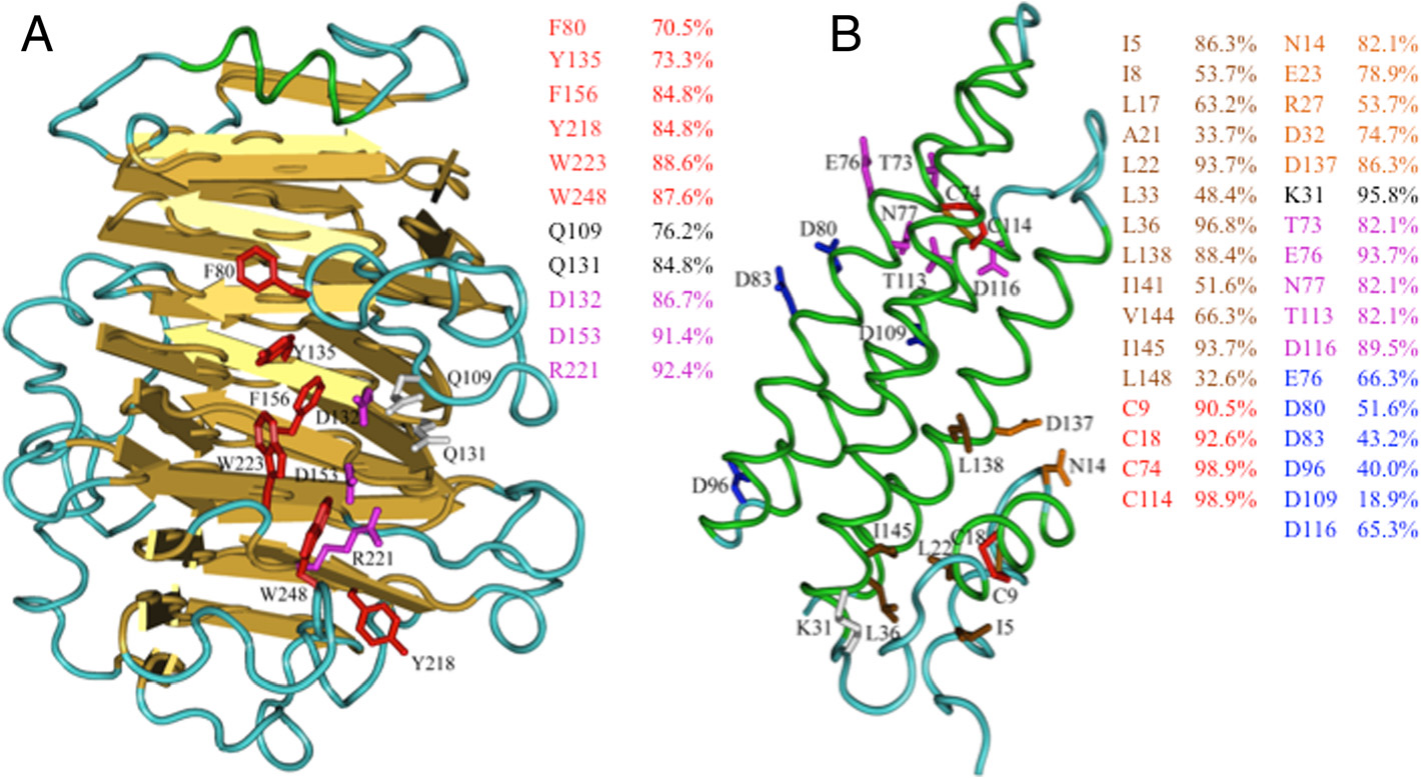
**Tertiary structures showing the conserved residues analyzed in relation to the reported structures.** Structures shown are for PME1_SOLLC **(A)** and PMEI_ACTDE **(B)**, PDB: 1XG2. For the residues involved in bundle-hairpin interface in PMEI_ACTDE only those residues with conservation higher than 80% are labeled in the structure.

We searched the predicted LuPMEs for conserved amino acid residues previously reported to be important for PME function. These amino acids were identified in a Type-1 PME from tomato (*Solanum lycopersicum*), (PME1_SOLLC, SwissProt P14280), and the positions listed here refer to that sequence [7]. Three residues are proposed to be catalytic residues: D132, D153, and R221. Two residues, Q109 and Q131, are believed to stabilize the intermediate that is formed after nucleophilic attack on the carboxylmethyl group. Finally, six aromatic amino acids at conserved positions are required for substrate binding (F80, Y135, F156, Y218, W223, and W248), and of these F80, Y135, and W223 are possible targets of the PMEIs. We searched the predicted LuPMEs for all eleven of the residues that have been proposed to be critical to PME function (Tables 1 and 2). The most highly conserved residues were the catalytic residues: D132, D153 , and R221, which could be identified in (91/105), (96/105), and (97/105) of the predicted LuPMEs, respectively (Figure 11A). The aromatic residues responsible for substrate binding were also highly conserved, where any aromatic residue is considered to be a conserved residue in comparison to the substrate binding positions defined in PME1_SOLLC (i.e. F80, Y135, F156, Y218, W223, and W248) . The most highly conserved of these were W223 (93/105) and W248 (92/105), and the conservation of other substrate binding residues was also high: F80 (74/105), Y135 (77/105), F156 (89/105), and Y218 (89/105). Among these aromatic residues there were three positions (F156, W223 and W248) in which the identity (and not merely similarity) of the aromatic amino acids was also highly conserved: at F156, F was found in 88/89 of the LuPMEs that had an aromatic residues at that position; at W223, 90/93 aromatic residues were W, and in W248 91/92 aromatic residues were W. F80, Y135, and W223 are responsible of substrate binding of the PMEs and also interact with the PMEI. F80 generated the highest number of contacts (17 in total) with the PMEI [7]. Out of all the aromatic residues, position F80 was the least conserved, followed by Y135, meanwhile W223 showed the highest conservation. This might imply that F80 and Y135 are not fundamental for binding to the substrate, as it might be W223, and on the other hand, lacking these residues may limit inhibition by the PMEIs.

### Conserved residues PMEI

PMEIs have four antiparalell alpha-helices (α1, α2, α3 and α4) arranged in an up and down topology, and three short alpha-helices (αa, αb, and αc) at the N-terminus [7]. Two groups have studied PMEI structure in detail. Di Matteo *et al*. [7] identified residues with important roles in the structure and activity of a PMEI from kiwi (*Actinidia deliciosa*; PMEI_ACTDE, SwissProt accession P83326), focusing on the interaction with a PME. They identified cysteine residues that generated disulfide bridges conecting helices αa and αb (C9 and C18), and helices α2 and α3 (C74 and C114). Furthermore, PMEI residues T73, E76, N77, T113, and D116 were found to allow interaction of the PMEI with three of the aromatic residues of the PME (F80, Y135, and W223). An acidic patch was formed by three conserved residues on both the α2 helix (E76, D80, and D83) and on the α3 helix (D96, D109, and D116). Finally, salt bridges occurred between the PMEI residues D116 and E76 and PME residues K224 and R81. Hothorn *et al.*[6] also studied the important residues for the PMEI activity and structure, using Arabidopsis PMEI-1 (PMEI1_ARATH, SwissProt accession number Q9LNF2) as a model. They identified a disulfide bridge connecting helices 5 and 6 (α2 and α3 in Di Matteo *et al. *[7]), formed by residues C71 and C111. They also identified a residue responsible for the N-terminal orientation (P28) that is located between the three N-terminal α-helices and the four α-helices towards the C-terminal; and several residues contributing to the bundle-hairpin interface.

We searched the predicted PMEIs of flax for the critical residues identified by Di Matteo *et al*. [7] and Hothorn *et al.*[6]. Our results (Table 3) are presented using as reference positions the mature PMEI_ACTDE protein. The residues in LuPMEIs with the highest conservation were C74 and C114 (both 94/95), which generate a disulfide bridge (Figure 11B). The conservation of the other two cysteines, C9 and C18, which stabilize the protein by hydrophobic interactions, was slightly lower, (86/95) and (88/95), respectively. The conservation (i.e. similarity, not necessarily identity) in the polar PMEI residues that interact with the aromatic PME residues, F80, Y135, and W223, was higher than 80% in all the cases: T73 (78/95), E76 (89/95), N77 (78/95), T113 (78/95), and D116 (85/95). 87.4% (83/95) of the PMEIs had conservation of at least 3 out of the 5 polar residues. On the other hand, the Aspartic acid (D), and Glutamic acid (E) residues that are predicted to be important in the generation of an acidic patch on alpha helices 2 and 3, had a low conservation (i.e. similarity, not necessarily identity), 47.5% on average. The conservation of the residues contributing to the bundle-hairpin interface was also low. Out of the 12 non-polar residues analyzed, only five were conserved (i.e. similarity, not necessarily identity) in more than 80% of the LuPMEIs, those are I5 (82/95), L22 (89/95), L36 (92/95), L138 (84/95), and I145 (89/95), the rest (I8, L17, A21, L33, I141, V144, and L148) were below 70% of conservation.

### Gene expression and conserved residues

We tested whether there was any correlation between the transcript expression evidence we obtained and the presence of the critical residues described above (Tables 1, 2 and 3). In general, genes that are not expressed may accumulate mutations, including mutations in residues critical to the normal function of the protein. We found that when we analyzed the 33 critical residues in the LuPMEIs as a group, the conservation of these residues among the expressed genes (73.2%) was significantly higher than in the genes for which transcript expression was not detected (63.9%) (Fisher’s Exact Test, p < 0.05). Taking together the 11 critical residues studied in the LuPMEs, we found that in the expressed genes, the level of conservation (86.8%) was significantly higher than the conservation observed in the non-expressed genes (75.3%) (Fisher’s Exact Test, p < 0.05). However when we individually analyzed the residues we found that in the LuPMEs only three out of the 11 residues (Q109, F156, and R221) showed significantly higher conservation in the expressed genes when compared to the genes without evidence of transcript expression (Fisher’s Exact Test, p < 0.05). In the remaining eight there was no significant difference (Fisher’s Exact Test, p > 0.05). The critical residue that showed the greatest change in conservation in relation to transcript expression was Q109, which was found in 83.1% of expressed LuPMEs, but only 57.1% of LuPMEs with no evidence of transcript expression. In the same way, we individually analyzed the LuPMEIs conserved residues, and found that only 5 of the 33 residues (L36, V144, C18, R27, and D83 ) have a significant higher conservation in expressed genes respect to non-expressed genes (Fisher’s Exact Test, p < 0.05). In the remaining 28 residues there was no significant difference (Fisher’s Exact Test, p > 0.05). The two greatest differences were observed in two of the residues contributing to the bundle-hairpin interface: V144 was conserved in 71.1% of expressed LuPMEs, but only 33.3% of non expressed genes, and R27 from 59.0% to 16.7%. Interestingly, the change in conservation was very different between the two pairs of cysteines of the PMEIs, which generate the disulfide bridges, C74 and C114 conservation changed both from 98.8% in expressed genes, to 100% in both, in non-expressed genes. Conversely, the conservation at positions C9 and C18 was reduced drastically, although not significantly in C9, from 92.8% and 96.4% in expressed genes, to 75.0% and 66.7%, respectevely in non expressed genes. This might indicate that there is more evolutionary pressure on residues C74 and C114, which indicates that they might be more important for the structure of the protein.

The residue at position K31(respect to PMEI_ACTDE [7]) or P28 (respect to PMEI1_ARATH [6]) has been reported to affect the orientation of the N-terminal of the PMEIs. We found 13 different amino acids at this position. There were three prevalent residues in this position, they were P (19/95), A (18/95), and I (17/95) residues. Hothorn *et al*. [6], found that when they mutated P28 to Ala in PMEI1_ARATH, the inhibitory activity of the protein diminished. So the 18 PMEIs with alanine could have a decline in activity.

## Conclusions

PMEs, regulated in part by PMEIs, modify cell and tissue properties by demethylesterification of pectins within cell walls and the middle lamella. Flax phloem fibers elongate intrusively by penetrating the middle lamella of the cells below and above them. This process requires the loosening of the middle lamella, the strengthening of the cell wall of the growing fiber, and then the creation of a new contact interface with the cell. In these processes, PMEs might be involved. Here we described 105 putative PMEs and 95 putative PMEIs in the flax genome, of which 77 and 83 genes, respectively, had evidence of transcript expression. The proportion of PMEs and PMEIs in the flax genome as a proportion of all predicted proteins was similar to most other dicots (Additional file 3: Figure S1). We defined a list of candidate genes that could play a role in fiber development (Table 4), their specific transcript expression in fiber containing tissues and mutant of these genes should be analyzed in detailed in future studies, and these genes area target for manipulation through reverse genetics.

## Competing interests

The authors declare that they have no competing interests.

## Authors’ contributions

DPL performed all experiments and analyses and wrote the original draft of this manuscript. MKD supervised experiments and analyses and edited the manuscript. Both authors read and approved the final manuscript.

## Supplementary Material

Additional file 1: Table S1 List of primers and TaqMan probes used for qRT-PCR. Click here for file

Additional file 2 FASTA formatted LuPME and LuPMEI sequences. Click here for file

Additional file 3: Figure S1 Percentage of PMEs and PMEIs, respect to the total number of proteins, in Embryophyta plants with available full genomes in Phytozome (version 9.1). Click here for file

Additional file 4: Figure S2 Maximum likelihood tree of PMEs in Flax and related species. The main groups, and important subgroup are shown. The homologous LuPMEs to PttPME1 and AtPME35 are shown with an arrow. Red: *Linum usitatissimum*; Purple (Me): *Manihot esculenta*; Blue (Rc): *Ricinus communis*; Orange (Pt): *Populus trichocarpa*; Green: *Arabidopsis thaliana*. 100 bootstraps and 2 search-replicates. Click here for file

Additional file 5: Figure S3 Maximum likelihood tree of PMEIs in flax and related species. The main groups, and subgroup D1 are shown. Red: *Linum usitatissimum*; Purple (Me): *Manihot esculenta;* Blue (Rc): *Ricinus communis;* Orange (Pt): *Populus trichocarpa;* Green: *Arabidopsis thaliana.* 100 bootstraps and 2 search-replicates. Click here for file

Additional file 6 Table S2 Genetic distance between possible paralogs in the LuPMEs and LuPMEIs gene families. The Kimura 2-parameter model was used to calculate the genetic distance, which was used to calculate the divergence time using t = K/2r, where t is time, K is the genetic distance, and r is the substitution rate, either 1.5 × 10^-8^ or 8.1 × 10^-9^. Click here for file
