# Supplementary figures and images for: Characterization and transcript profiling of the pectin methylesterase (PME) and pectin methylesterase inhibitor (PMEI) gene families in flax (Linum usitatissimum)
Source: BMC Genomics. 2013 Oct 30;14:742. doi: 10.1186/1471-2164-14-742 (PMC4008260; doi:10.1186/1471-2164-14-742)

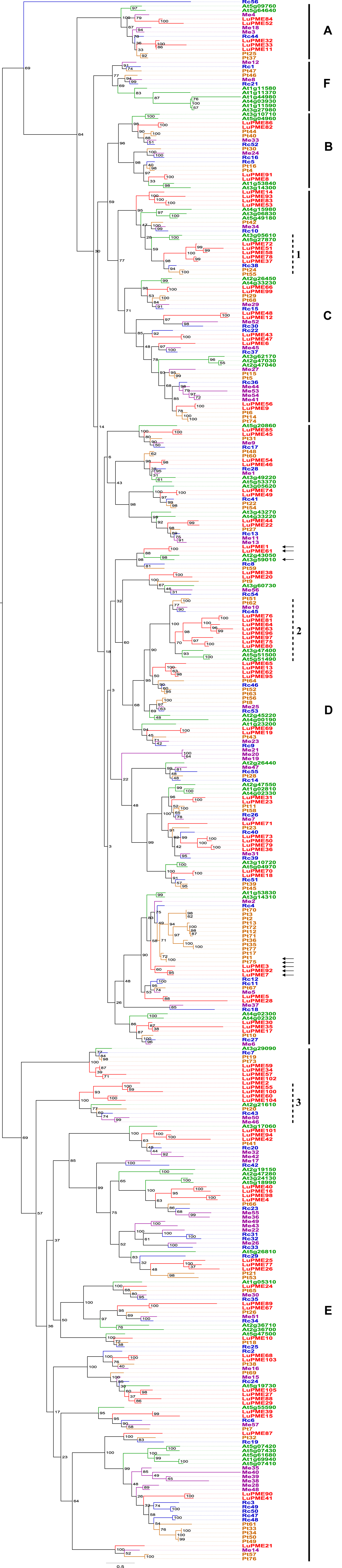

Supplement: Additional file 4: Figure S2 — Maximum likelihood tree of PMEs in Flax and related species. The main groups, and important subgroup are shown. The homologous LuPMEs to PttPME1 and AtPME35 are shown with an arrow. Red: Linum usitatissimum; Purple (Me): Manihot esculenta; Blue (Rc): Ricinus communis; Orange (Pt): Populus trichocarpa; Green: Arabidopsis thaliana. 100 bootstraps and 2 search-replicates. [file 1471-2164-14-742-S4.jpeg]

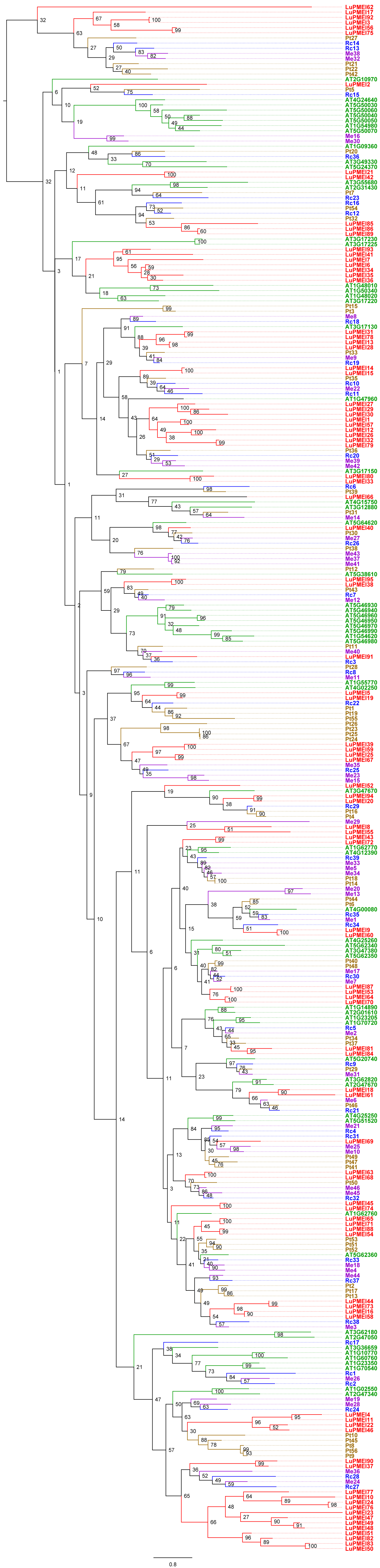

Supplement: Additional file 5: Figure S3 — Maximum likelihood tree of PMEIs in flax and related species. The main groups, and subgroup D1 are shown. Red: Linum usitatissimum; Purple (Me): Manihot esculenta; Blue (Rc): Ricinus communis; Orange (Pt): Populus trichocarpa; Green: Arabidopsis thaliana. 100 bootstraps and 2 search-replicates. [file 1471-2164-14-742-S5.jpeg]
