# Supplementary material for: Characterization and transcript profiling of the pectin methylesterase (PME) and pectin methylesterase inhibitor (PMEI) gene families in flax (Linum usitatissimum)
Source: BMC Genomics. 2013 Oct 30;14:742. doi: 10.1186/1471-2164-14-742 (PMC4008260; doi:10.1186/1471-2164-14-742)
Supplement: Additional file 6 Table S2 — Genetic distance between possible paralogs in the LuPMEs and LuPMEIs gene families. The Kimura 2-parameter model was used to calculate the genetic distance, which was used to calculate the divergence time using t = K/2r, where t is time, K is the genetic distance, and r is the substitution rate, either 1.5 × 10^-8^ or 8.1 × 10^-9^. [file 1471-2164-14-742-S6.docx]

|  |  | t = K/2r Millions | |
| --- | --- | --- | --- |
| Gene 1 | Gene 2 | r=1.50E^-08^ | r=8.1E^-09^ |
| LuPMEI3 | LuPMEI92 | 0.84 | 1.56 |
| LuPMEI14 | LuPMEI15 | 1.09 | 2.01 |
| LuPMEI20 | LuPMEI94 | 1.10 | 2.03 |
| LuPMEI13 | LuPMEI28 | 1.36 | 2.52 |
| LuPMEI48 | LuPMEI49 | 1.57 | 2.90 |
| LuPMEI4 | LuPMEI11 | 1.62 | 2.99 |
| LuPMEI63 | LuPMEI68 | 1.68 | 3.11 |
| LuPMEI56 | LuPMEI75 | 1.72 | 3.19 |
| LuPMEI50 | LuPMEI83 | 1.75 | 3.24 |
| LuPMEI45 | LuPMEI74 | 1.75 | 3.24 |
| LuPMEI9 | LuPMEI60 | 1.76 | 3.26 |
| LuPMEI53 | LuPMEI87 | 1.85 | 3.43 |
| LuPMEI16 | LuPMEI58 | 1.90 | 3.52 |
| LuPMEI81 | LuPMEI84 | 1.91 | 3.53 |
| LuPMEI86 | LuPMEI89 | 1.93 | 3.57 |
| LuPMEI24 | LuPMEI76 | 2.06 | 3.82 |
| LuPMEI54 | LuPMEI88 | 2.24 | 4.15 |
| LuPMEI64 | LuPMEI70 | 2.25 | 4.16 |
| LuPMEI25 | LuPMEI67 | 2.37 | 4.38 |
| LuPMEI32 | LuPMEI79 | 2.44 | 4.52 |
| LuPMEI1 | LuPMEI57 | 2.62 | 4.85 |
| LuPMEI44 | LuPMEI73 | 2.72 | 5.04 |
| LuPMEI5 | LuPMEI19 | 2.82 | 5.22 |
| LuPMEI31 | LuPMEI78 | 2.83 | 5.25 |
| LuPMEI65 | LuPMEI71 | 2.95 | 5.47 |
| LuPMEI38 | LuPMEI95 | 3.45 | 6.38 |
| LuPMEI43 | LuPMEI72 | 3.61 | 6.68 |
| LuPMEI12 | LuPMEI26 | 3.63 | 6.72 |
| LuPMEI39 | LuPMEI59 | 3.78 | 7.00 |
| LuPMEI6 | LuPMEI34 | 3.89 | 7.20 |
| LuPMEI33 | LuPMEI80 | 4.66 | 8.62 |
| LuPMEI22 | LuPMEI46 | 4.71 | 8.72 |
| LuPMEI37 | LuPMEI90 | 6.90 | 12.77 |
| LuPMEI21 | LuPMEI42 | 7.02 | 13.00 |
| LuPMEI29 | LuPMEI30 | 10.26 | 19.00 |
| LuPMEI18 | LuPMEI61 | 12.34 | 22.85 |
| LuPMEI35 | LuPMEI36 | 13.43 | 24.87 |
| LuPMEI41 | LuPMEI93 | 19.86 | 36.78 |
| LuPMEI8 | LuPMEI55 | 29.95 | 55.47 |
|  | Mean | 4.53 | 8.39 |
|  | Stdev | 5.72 | 10.59 |

|  |  | t = K/2r Millions | |
| --- | --- | --- | --- |
| Gene 1 | Gene 2 | r=1.50E^-08^ | r=8.1E^-09^ |
| LuPME41 | LuPME90 | 0.9 | 1.7 |
| LuPME103 | LuPME68 | 1.7 | 3.1 |
| LuPME18 | LuPME70 | 1.8 | 3.3 |
| LuPME66 | LuPME99 | 1.8 | 3.4 |
| LuPME56 | LuPME9 | 1.9 | 3.6 |
| LuPME19 | LuPME69 | 2 | 3.6 |
| LuPME43 | LuPME47 | 2 | 3.7 |
| LuPME46 | LuPME54 | 2 | 3.8 |
| LuPME1 | LuPME61 | 2 | 3.8 |
| LuPME12 | LuPME48 | 2.1 | 4 |
| LuPME49 | LuPME74 | 2.2 | 4 |
| LuPME8 | LuPME91 | 2.2 | 4 |
| LuPME45 | LuPME85 | 2.3 | 4.2 |
| LuPME15 | LuPME39 | 2.8 | 5.2 |
| LuPME20 | LuPME38 | 3.1 | 5.8 |
| LuPME82 | LuPME86 | 3.2 | 6 |
| LuPME22 | LuPME44 | 3.8 | 7 |
| LuPME52 | LuPME84 | 4.1 | 7.5 |
| LuPME23 | LuPME31 | 4.3 | 8 |
| LuPME67 | LuPME89 | 10.1 | 18.8 |
| LuPME28 | LuPME5 | 62.2 | 115.1 |
| LuPME11 | LuPME33 | 8 | 14.8 |
| LuPME53 | LuPME83 | 2 | 3.7 |
| LuPME37 | LuPME78 | 2.2 | 4 |
| LuPME51 | LuPME58 | 4 | 7.3 |
| LuPME75 | LuPME80 | 2.2 | 4 |
| LuPME76 | LuPME81 | 2.7 | 5.1 |
| LuPME63 | LuPME96 | 6.3 | 11.7 |
| LuPME62 | LuPME95 | 2.9 | 5.3 |
| LuPME50 | LuPME73 | 1.4 | 2.6 |
| LuPME36 | LuPME79 | 2.6 | 4.9 |
| LuPME7 | LuPME92 | 4.3 | 8 |
| LuPME17 | LuPME35 | 11.1 | 20.6 |
| LuPME34 | LuPME59 | 1 | 1.8 |
| LuPME102 | LuPME57 | 2.7 | 5 |
| LuPME104 | LuPME60 | 5.9 | 10.9 |
| LuPME100 | LuPME55 | 6.9 | 12.8 |
| LuPME42 | LuPME94 | 2.4 | 4.5 |
| LuPME16 | LuPME40 | 3 | 5.6 |
| LuPME4 | LuPME98 | 4.3 | 7.9 |
| LuPME26 | LuPME77 | 76.4 | 141.4 |
| LuPME105 | LuPME27 | 1.9 | 3.5 |
| LuPME29 | LuPME88 | 4.3 | 8 |
|  | Mean | 6.4 | 11.9 |
|  | St. dev. | 14.3 | 26.47 |
